# Endometrial decidualization status modulates endometrial microvascular complexity and trophoblast outgrowth in gelatin methacryloyl hydrogels

**DOI:** 10.1038/s44294-024-00020-4

**Published:** 2024-06-24

**Authors:** Samantha G. Zambuto, Hannah Theriault, Ishita Jain, Cody O. Crosby, Ioana Pintescu, Noah Chiou, Michelle L. Oyen, Janet Zoldan, Gregory H. Underhill, Brendan A. C. Harley, Kathryn B. H. Clancy

**Affiliations:** 1 Department of Bioengineering, University of Illinois Urbana-Champaign, Urbana, IL 61801, USA; 2 Department of Physics, Southwestern University, Georgetown, TX 78626, USA; 3 Department of Biomedical Engineering, University of Texas at Austin, Austin, TX 78712, USA; 4 Department of Molecular and Cellular Biology, University of Illinois Urbana-Champaign, Urbana, IL 61801, USA; 5 Department of Biomedical Engineering, Washington University in St. Louis, St. Louis, MO 63130, USA; 6 Center for Women’s Health Engineering, Washington University in St. Louis, St. Louis, MO 63130, USA; 7 Department of Obstetrics and Gynecology, Washington University School of Medicine, St. Louis, MO 63130, USA; 8 Carl R. Woese Institute for Genomic Biology, University of Illinois Urbana-Champaign, Urbana, IL 61801, USA; 9 Department Chemical and Biomolecular Engineering, University of Illinois Urbana-Champaign, Urbana, IL 61801, USA; 10 Cancer Center at Illinois, University of Illinois Urbana-Champaign, Urbana, IL 61801, USA; 11 Department of Anthropology, University of Illinois Urbana-Champaign, Urbana, IL 61801, USA; 12 Beckman Institute for Advanced Science & Technology, University of Illinois Urbana-Champaign, Urbana, IL 61801, USA

## Abstract

The endometrium undergoes rapid cycles of vascular growth, remodeling, and breakdown during the menstrual cycle and pregnancy. Decidualization is an endometrial differentiation process driven by steroidal sex hormones that is critical for blastocyst-uterine interfacing and blastocyst implantation. Certain pregnancy disorders may be linked to decidualization processes. However, much remains unknown regarding the role of decidualization and reciprocal trophoblast-endometrial interactions on endometrial angiogenesis and trophoblast invasion. Here, we report an engineered endometrial microvascular network embedded in gelatin hydrogels that displays morphological and functional patterns of decidualization. Vessel complexity and biomolecule secretion are sensitive to decidualization and affect trophoblast motility, but that signaling between endometrial and trophoblast cells was not bi-directional. Although endometrial microvascular network decidualization status influences trophoblast cells, trophoblast cells did not induce structural changes in the endometrial microvascular networks. These findings add to a growing literature that the endometrium has biological agency at the uterine-trophoblast interface during implantation. Finally, we form a stratified endometrial tri-culture model, combining engineered microvascular networks with epithelial cells. These endometrial microvascular networks provide a well-characterized platform to investigate dynamic changes in angiogenesis in response to pathological and physiological endometrial states.

To support tissue regrowth during the menstrual cycle and significant remodeling during pregnancy, the lining of the uterus, referred to as the endometrium undergoes rapid cycles of vascular growth, remodeling, and breakdown^1–3^. The endometrium is one of the only adult human tissues to undergo non-pathological angiogenesis^1–4^: endothelial cell-driven development of new vessels from existing blood vessels via elongation, intussusception, or sprouting^4,5^. During the menstrual cycle, angiogenesis occurs during three distinct phases: the proliferative phase, the secretory phase, and the menstrual phase^4–6^. The proliferative phase is characterized by the initiation of vessel growth^2,4,6^. In the secretory phase, the vessels continue to grow by branching, lengthening, and maturing^4,6^. Menstruation then induces vessel degeneration and endometrial shedding^6^. During menstruation, angiogenic processes also work to repair the superficial layer of the basal endometrium in preparation for the subsequent menstrual cycle^2,4^. During pregnancy, rapid angiogenesis also occurs in the endometrium immediately after trophoblast-uterine interfacing and before the formation of a placenta during pregnancy due to significant demands to support a growing placenta and eventual fetus^1,6^. Coordinated angiogenesis provides essential early support necessary to maintain these structures and their development^6–9^.

Sex steroid hormones progesterone and estrogen modulate endometrial angiogenesis and remodeling^2,3,6,10^. Progesterone controls vessel elongation, growth and coiling of spiral arterioles, and maturation of the subepithelial capillary plexus, whereas estrogen plays a key role in concert with the VEGF (vascular endothelial growth factor) family to control vascular remodeling^4–6,11^. These hormones also orchestrate a differentiation process known as decidualization. Decidualization is the process by which the endometrium prepares for a potential pregnancy by thickening and enhancing the tissue matrix for the incoming blastocyst^1,6,12^. During decidualization, vessels sprout and lengthen, the surface area of spiral arterioles increases, uterine glands undergo a secretory transformation, and specialized uterine natural killer cells increase in number^3,6^. Successful endometrial decidualization enables the endometrium to enter a period of receptivity that occurs during the mid-secretory phase of the menstrual cycle^2,6^. During this window, the blastocyst can attach to the endometrial epithelium and subsequently invade into the underlying stroma and vasculature^3,6^. Crosstalk between endometrial cells and trophoblast cells from the invading blastocyst are believed to modulate processes of invasion and dynamic vascular remodeling^2,6,7^. Endometrial decidualization is critical here, with reduced decidualization linked to a variety of pregnancy disorders such as preeclampsia, fetal growth restriction, and infertility^2,5–7^. For example, in the hypertensive pregnancy disorder preeclampsia, studies have demonstrated that in patients with severe preeclampsia, reduced decidualization subsequently led to impaired trophoblast invasion^2,6,7^. However, ethical considerations and technical limitations limit the ability to study these processes in vivo. As a result, much remains unknown regarding the role of sex steroid hormones on endometrial angiogenesis and vice versa.

Few models of endometrial vasculature exist. Most models of the endometrial vasculature either cannot recapitulate tissue biophysical properties (stiffness), do not use relevant human cell types in heterogenous cell cultures, or cannot be cultured long term (20+ days) days^13–17^. Engineered vasculature models have recently demonstrated promising results for mimicking vasculature in vitro for a wide range of applications. For example, Offeddu et al. and Haase et al. used perfusable vasculature in microphysiological microfluidic devices for quantifying transport through the endothelium and flow-mediated vessel remodeling^18–20^ and such model systems have also been used to study pregnancy-related vascular disorders, including placental vasculopathies. Engineered vasculature models can support three-dimensional culture of heterogeneous cell populations in biomaterials that can recapitulate tissue biophysical properties. Gelatin-based biomaterials are attractive materials for these types of studies for a variety of reasons. As a natural polymer derived from collagen, gelatin contains cell adhesion and degradation sites which allow for matrix remodeling by cells. Functionalization of gelatin by adding methacrylate groups to its amine-containing side groups results in the synthesis of gelatin methacryloyl, GelMA, which offers enhanced mechanical features that can be tuned to mimic *in vivo* biophysical properties, including the material properties of the nonpregnant and pregnant uterus^21,22^. Previous studies from our group and others have demonstrated that vessel networks can be cultured in GelMA hydrogels by encapsulating co-cultures of endothelial and stromal cells^21,23^.

Here, we develop and characterize models of an endometrial microvascular network embedded in GelMA hydrogels that display morphological and functional patterns of decidualization. We quantify shifts in microvascular network complexity, analyze soluble factor secretion of microvascular cultures, and assess matrix remodeling via basement membrane protein deposition and tight junction formation. Subsequently, we examine bidirectional communication, notably the role of trophoblast-secreted factors on microvascular network remodeling and the role of microvascular-secreted factors on trophoblast motility. Finally, we demonstrate a three-dimensional, stratified tri-culture model of the endometrium consisting of an epithelial culture overlaying the embedded microvascular networks. These microvascular networks replicate aspect of the in vivo endometrial microenvironment and can serve as a platform to study endometrial angiogenesis in a variety of endometrial states.

## Results

### Human endometrial microvascular endothelial cells (HEMEC) demonstrate angiogenic potential

We first assessed phenotypic markers and the ability to form vessel structures of human endometrial microvascular endothelial cells (HEMECs). HEMEC plated on 2D plates expressed CD31 (Supplemental Fig. S1A) and von Willebrand factor (Supplemental Fig. S1B). HEMECs plated on Matrigel transiently formed tubes, which indicted their angiogenic potential (Supplemental Fig. S1C). Taken together, these results suggest HEMECs express characteristic endothelial cell markers and they have the potential to form vessel-like structures in vitro.

### Endometrial microvascular networks in hydrogels can be cultured long term

**Fig. 1 |:**
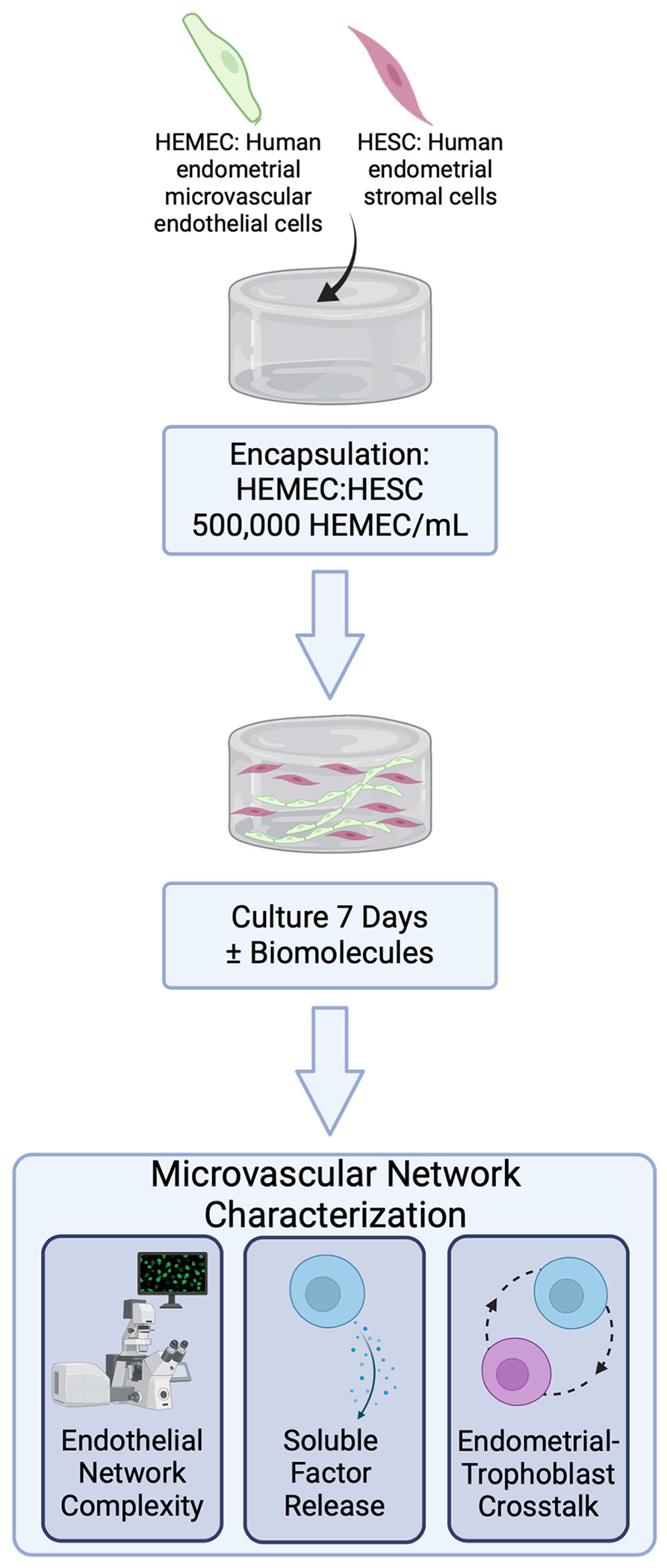
Development and characterization of endometrial microvascular networks. Encapsulated endometrial endothelial and stromal cells are co-cultured in gelatin methacryloyl hydrogels for 7 days and are subsequently analyzed for vessel network complexity, soluble factor secretion, and matrix remodeling. Created with Biorender.com.

**Fig. 2 |:**
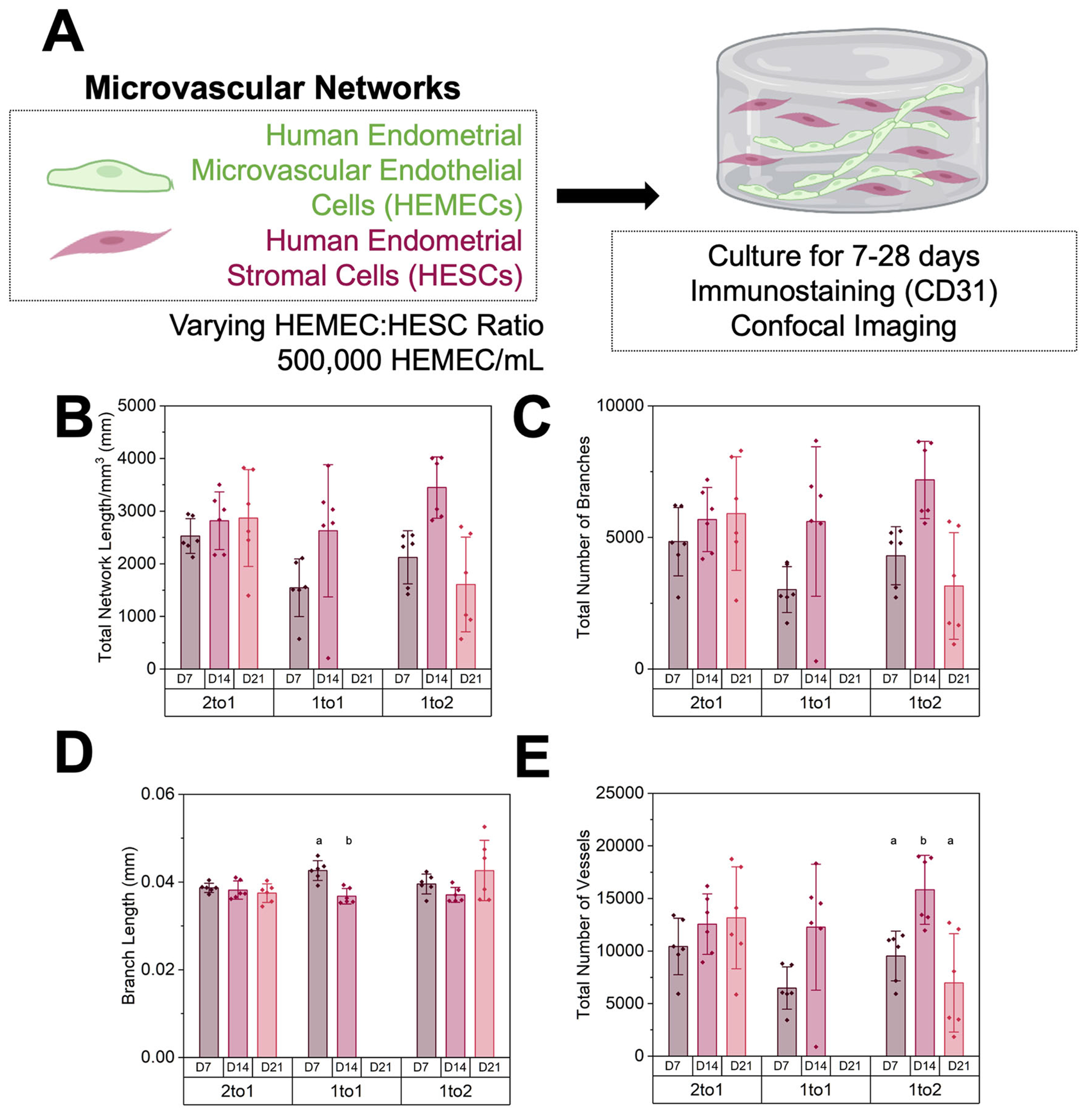
28-day culture of endometrial microvascular networks. **A** Experimental summary. **B** Quantification of total vessel length per mm^3^, **C** total number of branches, **D** average branch length, and **E** total number of vessels at days 7, 14, and 21 (*n* = 6 hydrogels per condition; 3 ROI imaged per gel and averaged) of varying endothelial to stromal cell ratios. Groups with different letters are statistically significantly different (*p* < 0.05; (D-T-test; *p* = 5.28 × 10^−4^; E-Welch’s ANOVA; *p* = 0.0061)) from each other. Data presented as mean ± standard deviation. Created with Biorender.com.

Next, we established endometrial microvascular networks and characterized key features of the culture system (Fig. 1). The longevity and stability of engineered microvascular networks were tracked for up to 28 days of culture in vitro. All microvascular networks contained a constant number of endothelial cells (500,000 cells/mL) but a variable number of stromal cells at ratios of 2:1 (250,000 stromal cells/mL), 1:1 (500,000 stromal cells/mL), and 1:2 (1,000,000 stromal cells/mL) endothelial:stromal cells (Fig. 2A). Notably, the ability to form stable microvascular networks was tightly tied to the ratio of endothelial and stromal cells. For the 1:1 endothelial:stromal laden hydrogels (Fig. 2B–E), total network length, branches, and vessels all increased from days 7 to 14 while branch length significantly decreased (T-test; *p* = 5.28 × 10^−4^; Fig. 2D); however, by day 21 all 1:1 hydrogels had disintegrated. Increasing the number of stromal cells (1:2 ratio) did not improve microvascular network stability. Here, total network length, branches, and vessels all increased from days 7 to 14 but then decreased from days 14 to 21 (Welch’s ANOVA; *p* = 0.0061; Fig. 2E), while branch length decreased between days 7 and 14 but increased between days 14 and 21. Although the 2:1 endothelial:stromal ratio microvascular network initially contained the least number of stromal cells, it was the most stable over time. The 2:1 endothelial:stromal ratio showed increasing metrics of total network length, branches, and vessels from days 7 to 21, while branch length appeared to remain consistent across all days with only a slight decrease over time (Fig. 2B–E). No culture remained stable through 28 days, with hydrogel degradation by the cells being the primary limitation of culture stability over long time periods.

### Ratio of endothelial to stromal cells affects microvascular network complexity more so than addition of VEGF

Soluble VEGF was added to culture media to determine if additional exogenous proangiogenic factors would affect microvascular network complexity (Supplemental Fig. S2A). We observed no significant differences in trends of metrics of microvascular network complexity as a result of VEGF inclusion (Supplemental Fig. S2B–E) and concluded that the endometrial microvascular networks do not require supplementation with additional proangiogenic factors. Based on these results and the results of the time course experiment, the 2:1 endothelial:stromal cell ratio microvascular network was used for all subsequent experiments because this condition resulted in the most consistent, stable networks over time.

### Microvascular network cultures deposit laminin and express tight junction marker ZO-1

Metrics of basement membrane protein deposition and expression of tight junction proteins were subsequently assessed for engineered endometrial microvascular networks. The engineered endometrial microvascular networks contained endothelial and stromal cells that elongated over time and expressed characteristic endothelial (CD31+ ; Supplemental Fig. S3A) and stromal (CD10+; Supplemental Fig. S3B) markers. Immunofluorescent staining demonstrated laminin, a common basement membrane protein, deposition by both cell types (Supplemental Fig. S4A–B). Additionally, ZO-1 expression was observed by day 7 of culture, suggesting the formation of tight junctions in our microvascular networks (Supplemental Fig. S4C). We deployed a simple colocalization script in MATLAB to quantify the degree of overlap between endometrial vessels (CD31+) and laminin/ZO-1 (Supplemental Fig. S4D–E). We calculated the average pixel intensity for both stains and calculated the degree of overlap by multiplying the binarized matrix of vessels by the binarized matrix of the proteins. Laminin/CD31 and ZO-1/CD31 both displayed approximately 30% signal overlap, suggesting that not only are laminin and ZO-1 expressed in engineered endometrial microvascular networks, but also they are expressed in close proximity to the endometrial microvascular networks formed within the GelMA hydrogels.

### Endometrial stromal cell decidualization status modulates endometrial microvascular network complexity

**Fig. 3 |:**
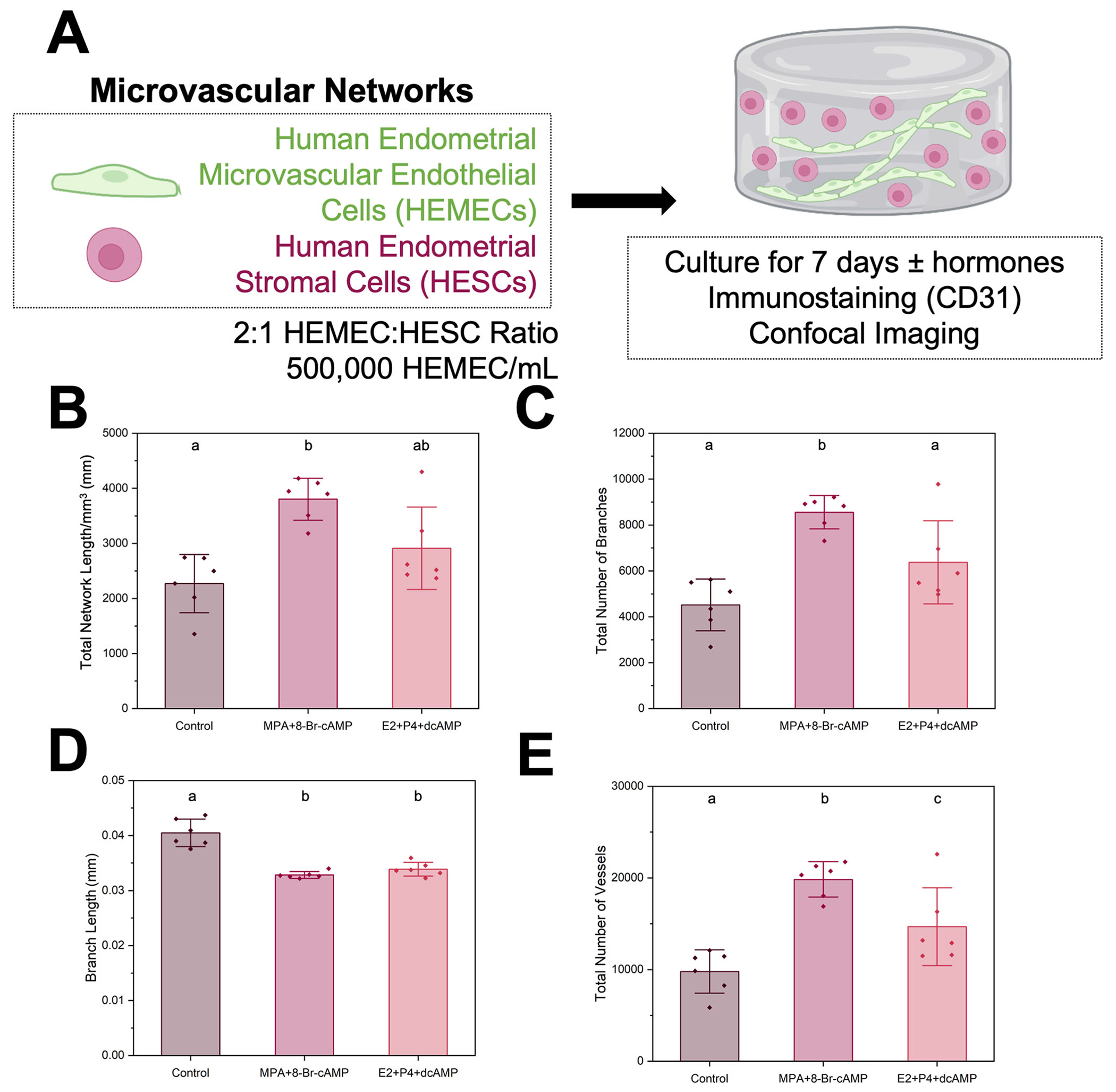
Stromal cell decidualization in endometrial microvascular networks. **A** Experimental summary. **B** Quantification of total vessel length per mm^3^, **C** total number of branches, **D** average branch length, and **E** total number of vessels for control and decidualized samples (*n* = 6 hydrogels per condition; 3 ROI imaged per gel and averaged). Two decidualization conditions were tested. The control condition contained no added decidualization hormones. Groups with different letters are statistically significantly different (*p* < 0.05) from each other (D-Welch’s ANOVA *p* = 0.00032). Data presented as mean ± standard deviation. MPA medroxyprogesterone acetate, Br-cAMP bromoadenosine cyclic AMP, E2 estradiol, P4 progesterone, dcAMP dibutyryl cyclic AMP. Created with Biorender.com.

Decidualization of the engineered endometrial microvascular networks was assessed in response to two decidualization protocols previously reported for two-dimensional cell culture^24–29^: exogenous addition of 1 μM synthetic progestin medroxyprogesterone acetate (MPA), 0.5 mM 8-bromodenosine 3’,5’-cyclic monophosphate (8-Br-cAMP) or 0.5 mM dibutyryl cyclic AMP (dcAMP)+10 nM estradiol (E2)+100 nM progesterone (P4) (Fig. 3A). We selected these decidualization methods because they are used interchangeably in the literature, and we were interested in assessing potential differences in network complexity of our cultures between using a synthetic progestin (MPA) versus not (P4). Morphological changes to the microvascular networks were first compared to a control condition (no decidualization hormones). Total network length, branches, and vessels increased in response to MPA decidualization condition compared to the control (Fig. 3B–E). Branch length was reduced for the MPA and P4 groups in comparison to control (Welch’s ANOVA and Games-Howell posthoc analysis; *p* = 0.00032; Fig. 3D). Total number of vessels increased for both decidualization conditions, with the MPA decidualization condition having the largest total number of vessels (Fig. 3E). Total network length and branches were not different between control and P4 decidualization conditions (Fig. 3B–C). Total network length and average branch length were not different between the MPA and P4 groups, but total branches were increased in the MPA group compared to the P4 group (Fig. 3B–E). Taken together, these results suggest decidualization status, as well as choice of decidualization hormone cocktail strongly influence microvascular network complexity in gelatin hydrogels, with decidualization broadly resulting in a denser network of smaller microvascular networks (increased number of shorter branches) and the strongest effect seen for decidualization with synthetic progesterone.

### Decidualization status strongly influences endometrial microvascular network secretome

**Fig. 4 |:**
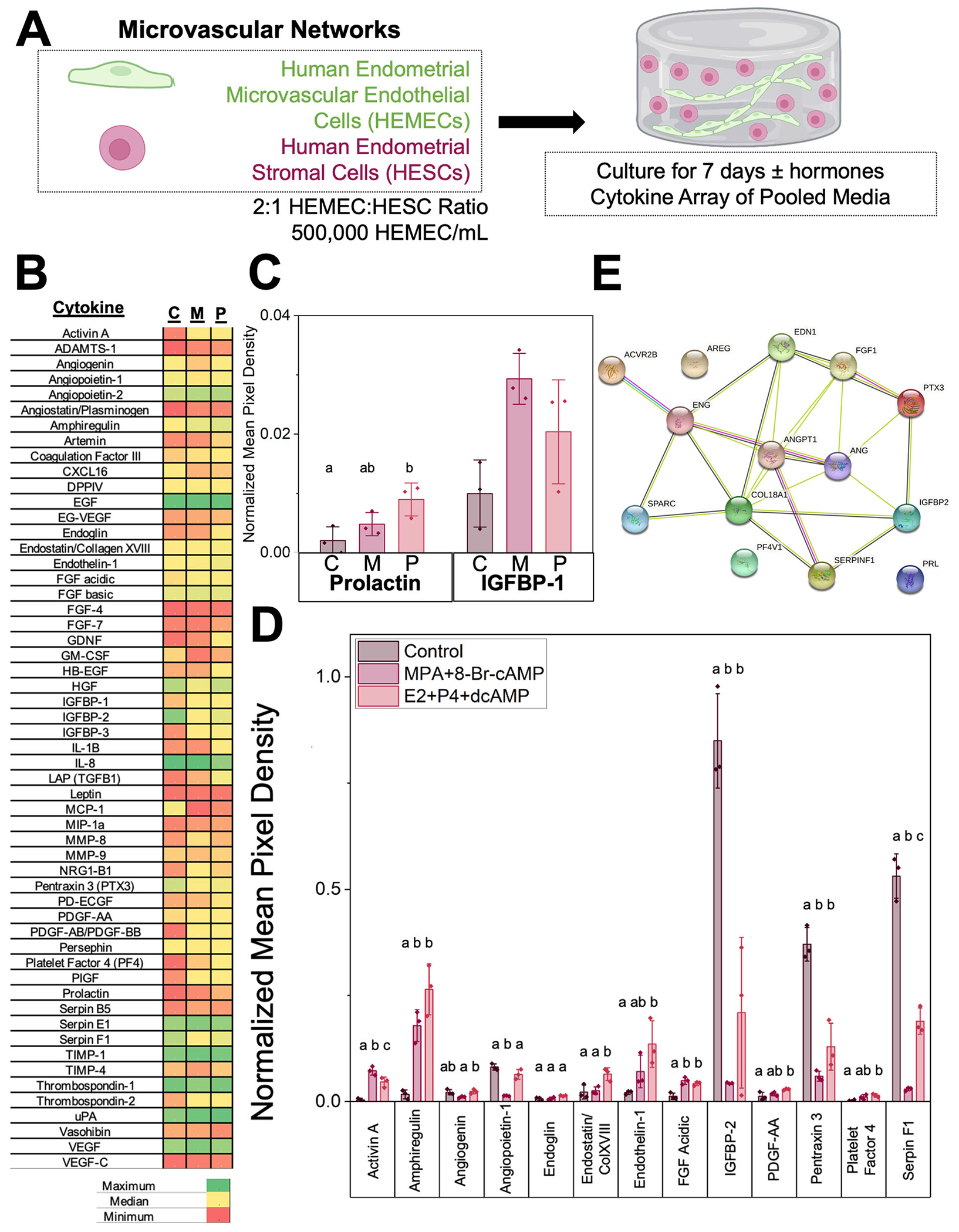
Cytokine secretion across control and decidualized microvascular network cultures. **A** Experimental summary. **B** Cytokine array normalized mean pixel density results C-Control, M-MPA, P-P4. **C** Normalized mean pixel density for characteristic decidual proteins Prolactin and IGFBP-1 across groups. C-Control, M-MPA, P-P4. **D** Normalized mean pixel density for statistically significantly different cytokines across groups. Groups with different letters are statistically significantly different (*p* < 0.05) from each other. Data presented as mean ± standard deviation. MPA: medroxyprogesterone acetate, Br-cAMP: bromoadenosine cyclic AMP, E2: estradiol, P4: progesterone, dcAMP: dibutyryl cyclic AMP. *N* = 3 hydrogels per condition. **E** STRING analysis of statistically significantly different cytokines in homo sapiens. Red line-fusion evidence. Green line-neighborhood evidence. Blue line-concurrence evidence. Purple line-experimental evidence. Yellow linetextmining evidence. Light blue line-database evidence. Black line-coexpression evidence. COL18A1-Collagen alpha-1 (XVIII). ANGPT1-Angiopoetin-1. FGF1-Fibroblast growth factor 1. AREG-Amphiregulin. PTX3-Pentaxin-related protein 3. EDN1-Endothelin-1. ENG-Endoglin. ANG-Angiogenin. PRL-Prolactin. ACVR2B-Activin receptor type-2B. PF4V1-Platelet factor 4 variant. IGFBP-2-Insulin-like growth factor binding protein 2. FGF1-Fibroblast growth factor 1. SERPINF1-Pigment epithelium-derived factor. Created with Biorender.com.

We subsequently examined biomolecular consequences of decidualization (control, decidualized-MPA, decidualized-P4) of microvascular networks via a cytokine array (quantifying mean pixel density of cytokine spots normalized to a positive control, Fig. 4A, B). We first compared the secretion of characteristic markers of decidualization prolactin and IGFBP-1 (insulinlike growth factor binding protein 1) (Fig. 4C). Prolactin and IGFBP-1 secretion increased for decidualized conditions compared to control, strongly indicating that stromal cells were decidualized in the presence of decidualization hormones. Subsequently, we performed statistical analysis across 55 human angiogenesis-associated proteins (statistical tests and p-values: Supplemental Figs. S5–6). We identified 14 proteins whose expression was significantly altered by microvascular network decidualization hormone cocktails: activin A, angiogenin, angiopoietin-1, amphiregulin, endoglin, endostatin/collagen XVIII, endothelin-1, FGF-1 (FGF acidic), IGFBP-2, Pentraxin 3 (PTX3), PDGF-AA, Platelet Factor 4 (PF4), Prolactin, and Serpin F1 (Fig. 4D; Table 1). We then used the STRING database to generate a qualitative network summary of predicted protein associations between the 14 proteins based on published literature evidence (Fig. 4E). The STRING network contained 14 nodes, 25 edges, 3.57 average node degree with an average local clustering coefficient of 0.495 and PPI enrichment *p* value < 1.0 × 10^−16^, and identified interactions between these factors, except for PF4, Prolactin, and Amphiregulin. Gene ontology analysis suggests these cytokines play critical roles in blood vessel development and branching, regulating endothelial cell proliferation, and epithelium branch elongation, with many of these cytokines associated with the basement membrane, ECM, extracellular space, and cytoplasmic vesicles.

### Microvascular network-secreted factors influence trophoblast invasion, but trophoblast-secreted factors do not affect microvascular network complexity

**Fig. 5 |:**
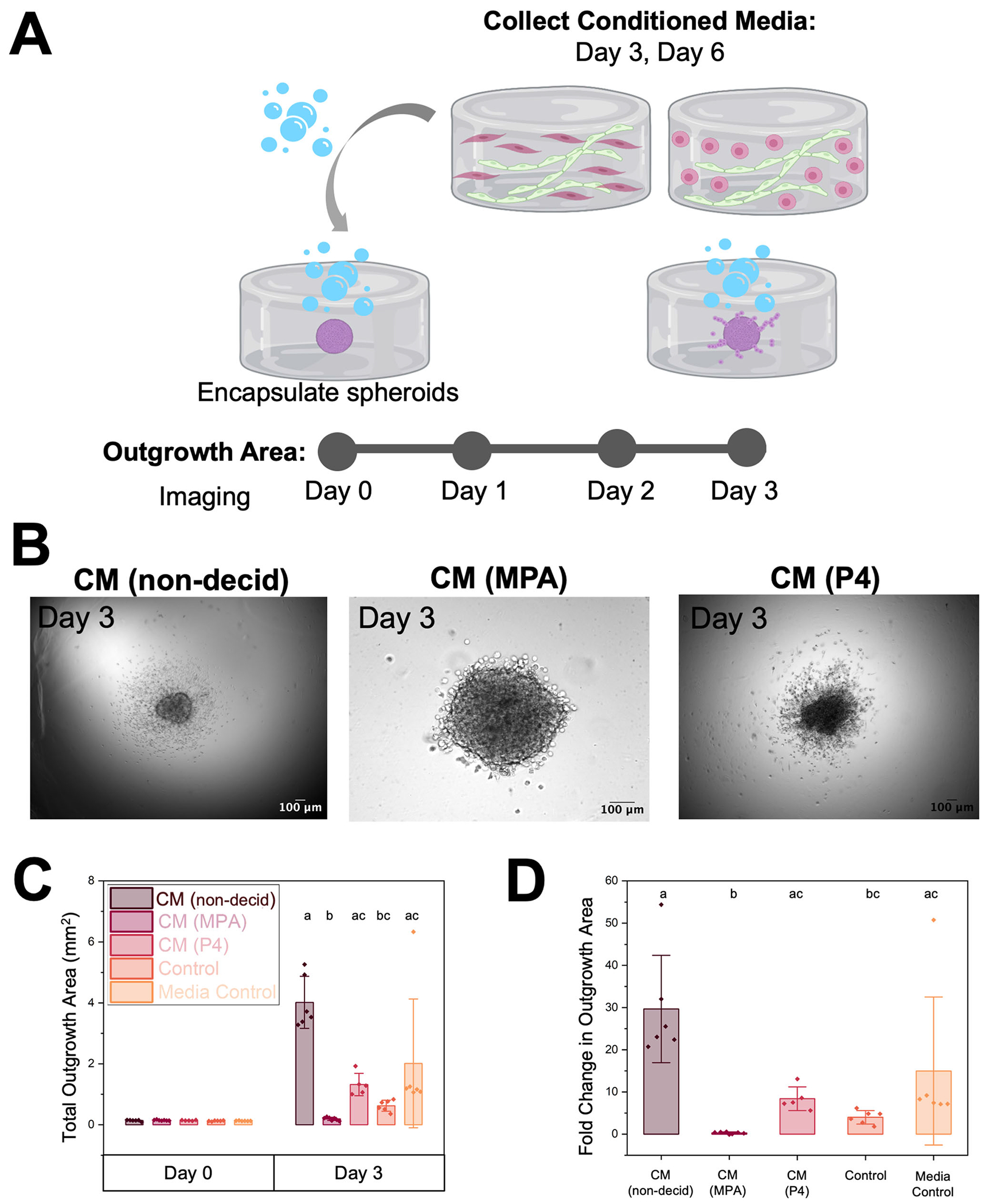
The effects of microvascular network conditioned media on trophoblast motility. **A** Experimental summary. **B** Representative images of trophoblast spheroid outgrowth on day 3 of culture. Scale: 100 μm. **C** Quantification of total outgrowth area (mm^2^) (Kruskal-Wallis ANOVA, *p* = 3.8 × 10^−5^) and **D** fold change in outgrowth area at Day 3 compared to Day 0 (encapsulation) (Kruskal-Wallis ANOVA, *p* = 3.8 × 10^−5^). Fold change was calculated by dividing the difference in outgrowth area between days 3 and 0 by the outgrowth area on day 0. Groups with different letters (Dunn’s Test) are statistically significantly different (*p* < 0.05) from each other. Data presented as mean ± standard deviation. CM Conditioned Media, Non-decid not decidualized (no hormones), MPA medroxyprogesterone acetate, P4 progesterone. Created with Biorender.com.

We then examined reciprocal crosstalk between endometrial microvascular networks and trophoblast cells. We first examined how microvascular network-secreted factors affect trophoblast motility. We quantified Swan71 trophoblast outgrowth area for 3 days in response to conditioned media collected from control or decidualized microvascular network hydrogel cultures using a previously described cell spheroid assay that quantifies total outgrowth area of spheroid (Fig. 5A). We collected conditioned media from non-decidualized (CM nondecidualized) or decidualized (CM MPA or CM P4) microvascular network hydrogel cultures and compared trophoblast invasion against two unique media controls (control or media control). We defined the control condition as a conventional trophoblast motility medium. The media control condition was a 1:1 mixture of trophoblast motility medium and microvascular network growth medium. This second control was used to replicate the media composition of the experimental groups, which contained a 1:1 mixture of trophoblast media and conditioned media collected from microvascular network cultures.

Outgrowth area was calculated over three days (Fig. 5B–C) and then fold change was calculated for each condition (Fig. 5D). Representative images of spheroid outgrowth are shown in Fig. 5B. By day 3, we observed significant differences in outgrowth area (Kruskal-Wallis ANOVA, *p* = 3.8 × 10^−5^) and fold change (Kruskal-Wallis ANOVA, *p* = 3.8 × 10^−5^) in outgrowth area (outgrowth at day 3 normalized to the same spheroid at day 0; Fig. 5C–D). Notably, trophoblast outgrowth was decreased in both decidualized conditions compared to the non-decidualized control condition. However, although both decidualization conditions resulted in decreased outgrowth area, conditions with MPA had lower outgrowth area compared to decidualized conditions with P4. Dunn’s post hoc analysis revealed four significant differences between outgrowth area groups: conditioned media (control-not decidualized) and conditioned media (MPA-decidualized) (*p* = 4.35 × 10^−5^), conditioned media (control-not decidualized) and control (*p* = 0.016), conditioned media (MPA-decidualized) and conditioned media (P4-decidualized) (*p* = 0.036), and conditioned media (MPA-decidualized) and media control (*p* = 0.011). Fold change was then calculated by normalizing initial spheroid outgrowth area to outgrowth area on day 3. The same trend observed in outgrowth area was also observed in fold change in outgrowth area, with *p* = 3.69 × 10^−5^, *p* = 0.016, *p* = 0.046, and *p* = 0.011, respectively (Dunn’s Test). Importantly, these findings demonstrate that not only microvascular network decidualization status but also mode of decidualization affect trophoblast outgrowth.

To verify the validity of our findings, we performed a control experiment for which we incubated Swan71 spheroid-embedded hydrogels with exogeneous decidualization hormone cocktails (Supplemental Figure S7). This control verified that exogeneous hormones reduce trophoblast motility compared to hormone-free cultures. Interestingly, the conditioned media from perivascular cultures supplemented with progesterone (P4) showed increased outgrowth area compared to the perivascular cultures supplemented with medroxyprogesterone acetate (MPA), which contrasted with the supplemental control experiment where there were no differences in trophoblast outgrowth area between the P4 and MPA groups. Interestingly, the secretome of the MPA-treated perivascular cultures showed the most quenched trophoblast invasion.

**Fig. 6 |:**
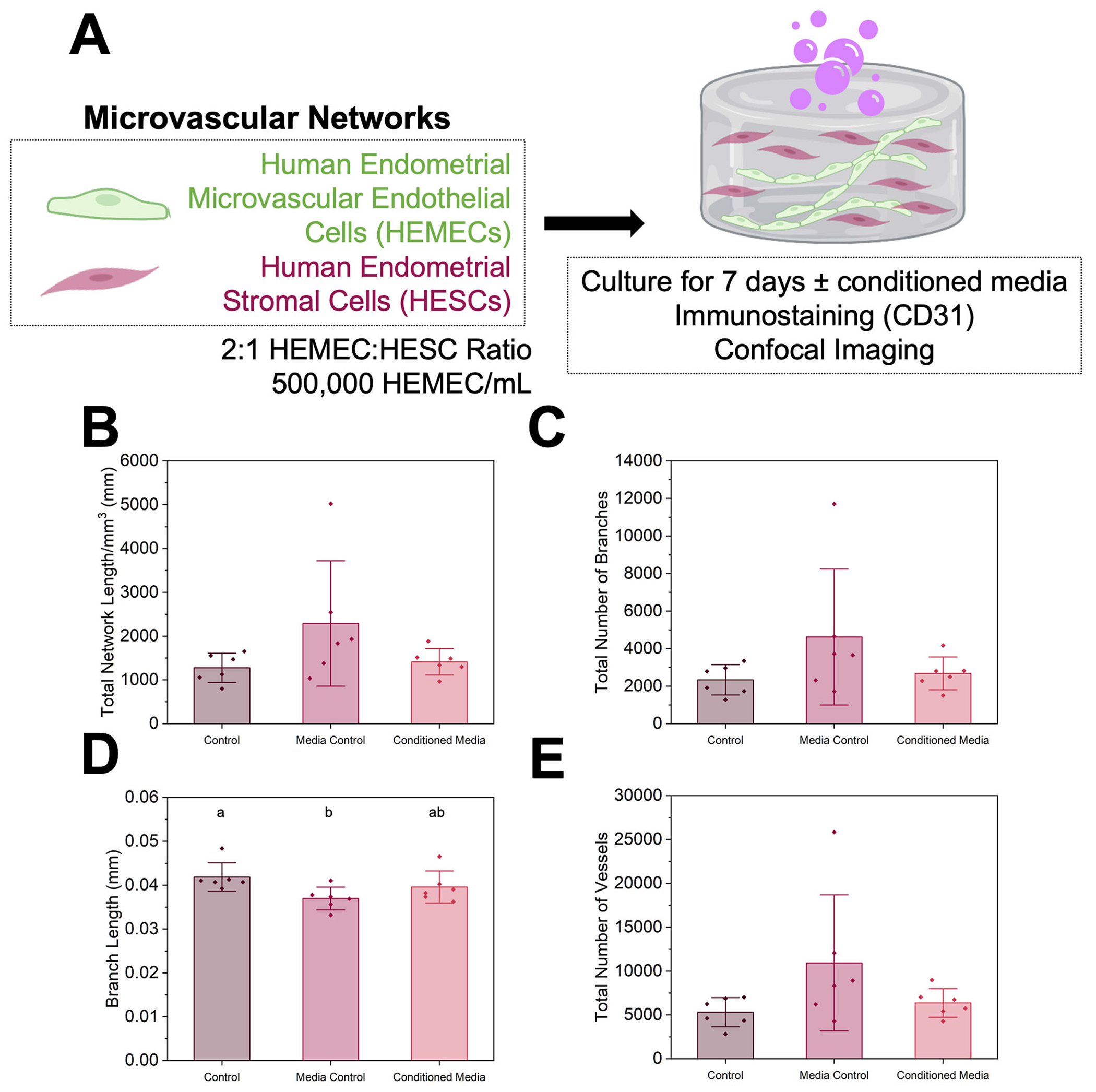
Effects of Swan71 trophoblast conditioned medium on endometrial microvascular network complexity. **A** Experimental summary. **B** Quantification of total vessel length per mm^3^, **C** total number of branches, **D** average branch length, and **E** total number of vessels for control, media control, and conditioned media samples (*n* = 6 hydrogels per condition; 3 ROI imaged per gel and averaged). Groups with different letters are statistically significantly different (*p* < 0.05; Kruskal-Wallis ANOVA with Dunn’s Test, *p* = 0.024) from each other. Data presented as mean ± standard deviation. Created with Biorender.com.

We then assessed whether and how trophoblast-secreted factors influenced microvascular network complexity. We quantified metrics of microvascular network complexity in the presence and absence of conditioned medium from Swan71 trophoblast cells (Fig. 6A). The control condition contained microvascular network growth medium, and the media control condition was a mixture of trophoblast and microvascular network growth medium consistent with the conditioned medium conditions. We observed no differences in total network length/mm^3^, total number of branches, and total number of vessels in the presence of conditioned media from Swan71 trophoblast cells (Fig. 6B, C, E). Although we did observe branch length decreased between the media control (50:50 media ratio) and control (Swan71 invasion media) conditions (Fig. 6D), this was likely due to increased serum content not depleted by cells.

### Endometrial tri-culture model: stratified endometrial epithelial culture overlaying microvascular networks

**Fig. 7 |:**
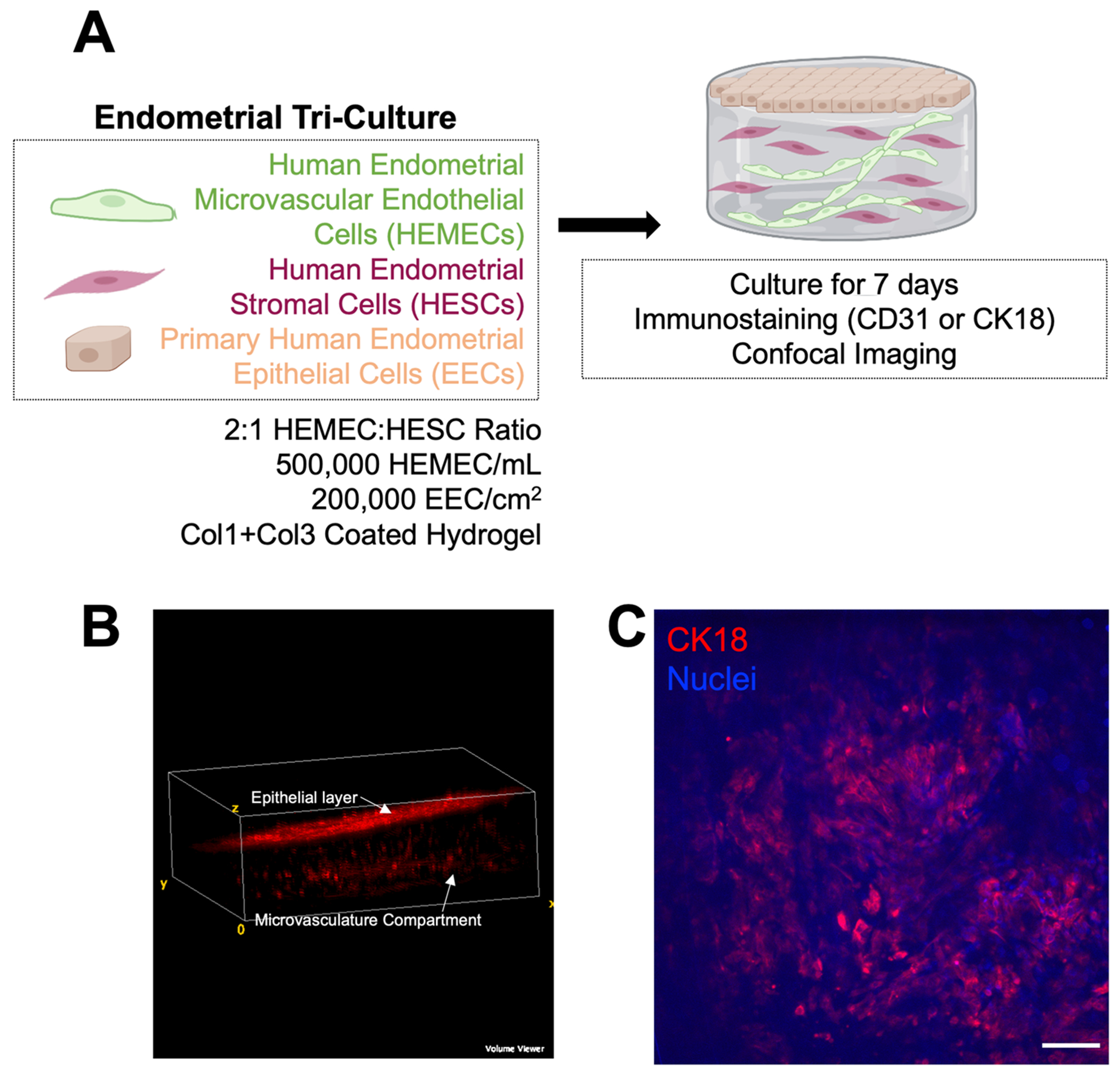
Fabrication of a stratified endometrial tri-culture model. **A** Experimental summary. **B** FIJI Volume Viewer maximum intensity projection showing phalloidin-stained cells with epithelial layer overlaying microvasculature compartment. **C** Maximum intensity projections of Z-stacks of tri-culture hydrogel cultures stained for cytokeratin 18 (CK18) to visualize epithelial cells. Scale bars: 100 μm. Created with Biorender.com.

Finally, as proof of concept for expanding the complexity of our microvascular network culture system, we fabricated an endometrial tri-culture to replicate features of the stratified endometrium *in vivo* by seeding primary endometrial epithelial cells on top of the 3D hydrogel microvascular network culture (Fig. 7A). Using phalloidin staining to visualize all three cell types simultaneously, we observed two stratified components: an epithelial layer overlying the microvascular network culture (Fig. 7B). We subsequently examined epithelial cell morphology and phenotype via immunohistochemistry for CK18, a marker of epithelial cell attachment (Fig. 7C). We observed regions of epithelial cells that positively express CK18, suggesting the epithelial culture is attached to the underlying microvascular network hydrogel.

## Discussion

Angiogenesis and vessel remodeling in the endometrium occur during the menstrual cycle as the tissue is rebuilt and differentiates to prepare for a potential pregnancy. Extensive remodeling of the existing vasculature occurs in response to the infiltration of trophoblast cells from the blastocyst to provide blood flow to the growing fetus and placenta. Here, we report the creation of a three-dimensional endometrial microvascular network embedded in gelatin methacryloyl hydrogels. The endometrial microvascular networks demonstrate the capacity to exhibit morphological and biomolecular signatures of decidualization in response to exogenous hormones. These microvascular networks provide a platform to examine reciprocal crosstalk between the microvascular networks and trophoblast cells. Further, the engineered microvascular networks can be combined with our recently reported methods for endometrial epithelial cell culture to establish a stratified tri-culture endometrial model consisting of primary endometrial epithelial cells overlaying the microvascular networks^30^. Combined, these elements of a stratified endometrial model offer significant potential to gain mechanistic insight into endometrial remodeling and changes in endometrial vascular networks in response to decidualization.

Most existing in vitro models of vasculature utilize human umbilical vein endothelial cells (HUVECs) as their endothelial cell source due to their wide availability, capacity to be passaged numerous times, and excellent ability to form vessels in vitro^31^. However, HUVECs are derived from umbilical cords and are not tissue-specific, which calls into question the use of such cells for the development of tissue-specific vasculature models. Here, we utilize HEMECs as an endothelial cell source to create an endometrial-specific model of microvascular networks. We also used human endometrial stromal cells (HESCs) as an endometrial stromal cell source. The addition of a stromal population to endothelial cells encourages and supports formation of endothelial networks for long-term culture^32–34^. Without stromal cells, endothelial cells form endothelial structures that last only transiently and fall apart over time, consistent with our results from our Matrigel tube formation assay. We identified 2:1 ratio of endothelial to stromal cells allowed for 20+ days of culture.

Our studies suggest that reduced initial stromal cell density may be more conducive to microvascular network stability, likely due to the contractile nature of stromal cells, which can cause the hydrogel cultures to contract and disintegrate over time, a result consistent with other reports in the literature^35,36^. One limitation of our model system is that there is high variability for some of the measured metrics of network complexity. Although the number of vessels, branches, and total network length can be variable across experiments, the average branch length is consistent across all experiments and all replicates. We judged variability based on the average branch length and sought to keep this variable consistent so that the individual branches of the networks were similar. Variability in vessel parameters is to be expected because of the highly dynamic nature of these types of systems. Previous studies have demonstrated that spatial geometry and vessel parameters can be heterogeneous for certain cell types and vary tissue-to-tissue^37^.

We then demonstrate that endometrial microvascular networks do not require exogenous pro-angiogenic factors (VEGF) to promote network formation. Endometrial microvascular cultures also express basement membrane protein deposition and tight junction markers. Our microvascular network cultures demonstrate similarities to the native endometrium. The average vessel length per branch point was determined to be approximately 100-200 μm across the menstrual cycle, and this value varies depending on cycle phase^38^. Although our average branch values were less than these *in vivo* values, we observed significant differences in microvascular network complexity with the addition of hormones. This indicates that microvascular networks formed in vitro from endometrial-derived endothelial and stromal cells are hormone-responsive. Given emerging literature seeking to better define population variation in hormone concentration across the menstrual cycle, an engineered endometrial microvascular network culture may provide the opportunity to quantify shifts in vessel complexity in response to exogenous hormone signals representative of discrete menstrual cycle phases. Future work to assess lumen formation and patent vessels would allow for the creation of perfusable networks to assess molecule transport and vessel perfusion in the context of pregnancy.

We subsequently sought to determine how the decidualization status of stromal cells affects the microvascular networks. Decidualization is necessary and critical to prepare the endometrium for a potential pregnancy^6^. Therefore, recapitulating such processes may be important for an endometrial model system used to study implantation events. For these studies, we chose two decidualization protocols commonly used in the literature^24–29^. One employs the synthetic progestin medroxyprogesterone acetate (MPA), and the other uses progesterone (P4). These methods are often used interchangeably but we were interested in assessing potential differences in network complexity of our cultures between using a synthetic progestin versus not.

Across our studies, we observed differences in the effects of these two protocols in our microvascular networks, notably the effects of the P4-decidualized condition did not seem to increase total network length/mm^3^, total vessels, and total branches as much as the MPA-decidualized condition. Analysis of our microvascular networks in the absence and presence of decidualization hormones revealed an increasing trend for total network length/mm^3^, total number of branches, and total number of vessels with the addition of hormones. Interestingly, branch length significantly decreased with the addition of hormones. Observations from human specimens demonstrate that the endometrial spiral arterioles grow, lengthen, and coil during the secretory phase of the menstrual cycle (phase when decidualization occurs)^4^. These data are consistent with some our observations, further demonstrating that this model system captures some endometrial physiologic responses in vitro.

Next, we analyzed shifts in the microvascular network secretome in response to decidualization. Our analysis of the microvascular network secreted factors detected 14 cytokines with statistically significant differences between the conditions: Activin A (Gene ID 3624), Amphiregulin (AR; Gene ID 374), Angiogenin (ANG; Gene ID 283), Angiopoeitin-1 (Ang-1; Gene ID 284), Endoglin (ENG; CD105; Gene ID 2022), Endostatin/Collagen XVIII (Gene ID 80781), Endothelin-1 (ET-1; Gene ID 1906), FGF acidic (FGF-1; Gene ID 2246), IGFBP-2 (Gene ID 3485), PDGF-AA (Gene ID 5154), Pentraxin 3 (PTX3; TSG-14; Gene ID 5806), Platelet Factor 4 (PF4; CXCL4; Gene ID 5196), Prolactin (Gene ID 5617), and Serpin F1 (PEDF; Gene ID 5176). These 14 proteins can be broadly characterized into proteins associated with angiogenesis, vessel stabilization, and vessel maturation (Angiogenin, Angiopoietin-1, Endostatin/Collagen XVIII, FGF-1, PDGF, PTX3, Serpin F1), proteins relevant to endometrial function and receptivity (Amphiregulin, Endoglin, Endothelin-1, IGFBP-2, PF4); and, proteins relevant to decidualization and stromal cells (Activin A, Angiogenin, PDGF, Prolactin).

Angiogenin, Angiopoietin-1, Endostatin/Collagen XVIII, FGF-1, PDGF, PTX3, and Serpin F1 are related to angiogenesis, vessel stabilization, and vessel maturation. The overall presence and corresponding changes in these factors in non-decidualized vs. decidualized conditions indicate that decidualization hormones directly impact endometrial angiogenesis. For example, low levels of Ang-1 are found in endometrial stromal fibroblasts, and this decreases over the menstrual cycle^39^. We observed a significant decrease in Ang-1 in one of the decidualization conditions compared to the control. Although the second condition did not show a significant decrease, it did appear to be slightly lower than the control values. We also observed increases in endostatin and FGF-1 in decidualized conditions, which could indicate vessel maturation, stabilization, and angiogenesis^39–41^. Additionally, decidualization may induce proliferation or motility of cells because these conditions demonstrated increased expression of PDGF-AA, a growth factor shown to have roles in cell proliferation, angiogenesis, inflammation, and tissue repair^42^.

Amphiregulin, Endoglin, Endothelin-1, IGFBP-2, and PF4 are proteins relevant to endometrial function and receptivity. The secretion of these factors suggests our microvascular network cultures demonstrate relevant endometrial cell behavior. For example, amphiregulin and CXCL4 were both shown to have increased secretion in decidualized samples. Amphiregulin is a member of the epidermal growth factor (EGF) family and has a role in uterine receptivity and blastocyst attachment^43^. Amphiregulin has been found in the luminal epithelium at the site of blastocyst apposition and its expression is correlated with an increase in progesterone levels and blastocyst attachment^43^. Our data also demonstrate this trend, with significantly increased levels of Amphiregulin in decidualized microvascular cultures containing increased progesterone. CXCL4 is also regulated by progesterone withdrawal, which suggests it likely has a role in endometrial repair following menses^44^. Our results were consistent with these data: CXCL4 secretion was increased in decidualized microvascular cultures.

Activin A, Angiogenin, PDGF, and Prolactin are proteins relevant to decidualization and stromal cells. Activin A is produced in high concentrations by decidualized stromal cells and interacts with matrix metalloproteinases (MMPs) to promote matrix remodeling in the decidual response^45,46^. Our data were consistent with these previous observations: we observed significantly increased levels of Activin A in decidualized microvascular networks compared to control.

Although much of our data are consistent with the literature, differences could be due to donor variability or the use of cell lines instead of primary cells^47–49^. For example, previous data using endometrial epithelial cells noted donor-to-donor differences in epithelial cell behavior, so we would suspect to see potential differences in the use of donor-derived HEMECs as well^47^. This could be further explored using additional cells from more donors. Furthermore, signaling from other cells in the endometrium (e.g., epithelial cells, natural killer cells, immune cells, etc.) could alter the secretome of stromal and endothelial cells, which could account for some of these differences^3,5,50,51^. Expanded studies using additional endometrial cell types could begin to probe these differences and glean additional insights into the secretome of other endometrial cells.

Next, we were interested in the constellation of signals from decidualization since external stimuli cause decidualization through a cascade of mechanisms. This includes many other factors aside from the hormones, including what the stromal and endothelial cells secrete in the presence of hormones, how the hormones are hydrolyzed or degraded by the cells, and what is retained in the media overall. Our objective for this experiment was to determine how the decidual secretome of endometrial microvascular networks affects trophoblast invasion. The conditioned media from non-decidualized microvascular networks increased trophoblast motility compared to the other tested conditions.

Interestingly, conditioned media taken from the two decidualization conditions (MPA- and P4-decidualized) induced differential responses regarding trophoblast motility. Conditioned media from the P4-decidualized microvascular networks increased trophoblast motility compared to the non-decidualized control (Swan71 invasion medium); however, the MPA-decidualized conditioned media induced less outgrowth compared to the non-decidualized control. Critically, these data show that endometrial microvascular network decidualization status influences the activity of trophoblast cells via secreted factors.

To verify the validity of these findings, we performed a control experiment for which we incubated Swan71 spheroid-embedded hydrogels with exogeneous decidualization hormone cocktails and determined that exogeneous hormones reduce trophoblast motility compared to hormone-free cultures. The conditioned media from perivascular cultures supplemented with P4 showed increased outgrowth area compared to the perivascular cultures supplemented with MPA; however, Swan71 spheroid-embedded hydrogels supplemented with exogeneous decidualization hormone cocktails had no differences in trophoblast outgrowth area between the P4 and MPA groups. As such, the results strongly suggest that the microvascular network secretome is responsible for the differential changes in the outgrowth area observed in this experiment. Another important conclusion is that the secretome of the MPA-treated perivascular cultures showed the most quenched trophoblast invasion, which should be a consideration when using medroxyprogesterone acetate as a decidualizing agent.

Although conditioned media (MPA- and P4-decidualized) from decidualized microvascular networks only induced a moderate increase in trophoblast motility, this only represents a single condition of hormone stimulation targeting initial decidualization events. There is a significant opportunity for future efforts to examine how trophoblast motility changes in response to a dynamic secretome based on hormone concentrations representative of greater shifts across the menstrual cycle. Notably, treatment of microvascular networks with Swan71 trophoblast conditioned medium did not change the majority of markers used to assess microvascular network complexity; however, we did observe decreased branch length in the media control condition (50:50 ratio Swan71 invasion medium and endothelial growth medium) compared to the control condition (Swan71 invasion medium). This could be because the Swan71 unconditioned growth medium contains less proangiogenic factors compared to endothelial growth media.

We note that, in our system, trophoblast-microvascular signaling was not bi-directional. Although endometrial microvascular network decidualization status influences the activity of trophoblast cells, trophoblast cells do not appear to influence structural changes in the endometrial microvascular network cultures. This finding has interesting implications as to the control of early stages of trophoblast motility from the endometrium but no marked early changes in its microvascular architecture in response to initial trophoblast implantation. These findings add to a growing literature that the endometrium has biological agency in the uterine-trophoblast interfacing process during implantation^52–54^; this is a fascinating topic that is worthy of more in depth studies.

Finally, we demonstrate the creation of a stratified endometrial tri-culture consisting of an endometrial epithelial layer overlaying the embedded microvascular networks. This experimentation serves as proof of concept for creating endometrial model systems of increasing complexity and demonstrating the feasibility of replicating endometrial tissue architecture. Our methodology offers a simpler way to attach cells to gels by simply adding extracellular matrix molecules on top of the gels instead of directly modifying the polymer backbone with these features, which would affect all the cells within the hydrogel instead of only the cells on top. Our work herein demonstrates an endometrial model of increased complexity compared to existing models that incorporates three endometrial cell types in one model system. We chose collagen I and collagen III as the basement membrane layer as our prior work demonstrated this combination of ECM biomolecules resulted in the best epithelial cell attachment^55^. Our model expands upon existing stratified endometrial model systems because we have added additional complexity by not only including stromal cells but also endothelial cells to create embedded microvascular networks rather than only an embedded stroma^47^.

Future work will focus on quantification of microvascular network metrics for the tri-culture as well as on the hormonal work to determine how decidualization affects not only the microvascular network compartment but also the epithelial layer. Additional opportunities for these studies include the development an endometrial microvascular network using primary endometrial stromal cells. HESCs are the most widely used cell line for endometrial stromal cells; however, as hTERT-immortalized cells, they may not mimic endometrial stromal cells as closely as primary cells could. The use of patient-derived cells could ameliorate this challenge and provide additional insights into endometrial perivascular function. Similarly, although we used a widely studied trophoblast cell line (Swan71), we recognize the limitations of using a cell line over primary cells. We chose to use Swan71 cells over other trophoblast cell lines because they are not derived from choriocarcinoma, are immortalized using hTERT to produce trophoblast cells of normal karyotype and phenotype, and have been shown to express cytokeratin 7, human leukocyte antigen-G (HLA-G), human chorionic gonadotropin, and fetal fibronectin similarly to primary trophoblast cells. Nonetheless, primary cells or stem cells would be the most physiologically relevant option. Additionally, this work considers sex steroid hormone profiles from one point in the menstrual cycle. Ongoing work is looking to quantify metrics of microvascular network formation across the entire menstrual cycle to assess cyclic microvascular network formation and cell-mediated remodeling, including MMP secretion or cathepsin activity. As studies in humans have shown variation in menstrual cycle length and hormone profiles^56,57^, there is a significant opportunity to use this platform as a route to explore patient variation via the incorporation of different hormone profiles.

In conclusion, we describe the creation of endometrial microvascular networks embedded in GelMA hydrogels. Engineered endometrial microvascular network cultures display hormone responsiveness, including variation in network complexity and secretion of soluble factors. We show a model of unidirectional signaling; although microvascular network conditioned medium increased trophoblast motility in spheroid motility assays, trophoblast conditioned medium showed a limited effect on microvascular network complexity. Finally, we describe a stratified endometrial tri-culture model consisting of an endometrial epithelium overlaying the embedded microvascular networks. Tissue engineering models such as these not only provide novel platforms for assessing endometrial function but also allow us to probe questions regarding implantation that are currently impossible to answer in humans due to ethical constraints, challenging time points, and lack of imaging modalities. With the creation of these platforms, we hope to provide researchers with novel technologies that can further the field of uterine health.

## Methods

### Experimental design

The objective of this study was to design, characterize, and implement an engineered model of endometrial vasculature. Using gelatin methacryloyl hydrogels, we create a co-culture system of endometrial endothelial and stromal cells, which we call the microvascular network, and subsequently assess response to sex steroid hormones and trophoblast secreted factors.

### Synthesis and fabrication of gelatin methacryloyl (GelMA) hydrogels

GelMA was synthesized, dialyzed, lyophilized, and was found to have a degree of functionalization of 57%, determined via ^1^H-NMR^58–60^. Prior to cell culture experiments, lyophilized GelMA was sterilized for 30 minutes under UV light. Hydrogels were fabricated using a solution consisting of lyophilized GelMA (5 wt%) dissolved at 37 °C in phosphate-buffered saline (PBS; Lonza 17-516 F) and combined with 0.1% w/v lithium acylphosphinate (LAP) as a photoinitiator. Hydrogels were cast into a custom Teflon mold (well size: 5 mm diam., 1 mm height, 20 μL solution per well) and subsequently polymerized under UV light (λ = 365 nm, 7.14 mW cm^−2^; AccuCure Spot System ULM-3-365) for 30 s.

### Cell culture and maintenance

#### Human endometrial microvascular endothelial cell culture.

Human endometrial microvascular endothelial cells (HEMEC; ScienCell #7010) were maintained as per the manufacturer’s instructions in phenol red-free Endothelial Cell Medium (ScienCell #1001-prf) supplemented with an endothelial cell growth supplement (ScienCell #1052), 5% charcoal-stripped fetal bovine serum (Sigma-Aldrich F6765), and 1% penicillin/streptomycin (ThermoFisher 15140122). Charcoal-stripped fetal bovine serum was used to reduce the steroid hormone concentrations in the cell medium. HEMECs were cultured on bovine plasma fibronectin (ScienCell #8248) coated vessels. HEMECs were used experimentally no more than 5 passages from purchase. HEMECs were cultured in 5% CO_2_ incubators at 37 °C. Routine mycoplasma testing was performed using a MycoAlert^™^ Mycoplasma Detection Kit (Lonza). Cell ancestry information (e.g., racial and ethnic background, age, gender identity) was not provided by the vendor although the cell ancestry may affect cellular behavior and response^48,49^.

#### Human endometrial stromal cell culture.

Human endometrial stromal cells (HESC; ATCC^®^ CRL-4003) were maintained as per the manufacturer’s instructions in custom phenol red-free DMEM/F-12 (based on Sigma #D 2906) supplemented with 1% ITS+ Premix (Corning 354352), 500 ng/mL puromycin (Millipore Sigma P8833), 10% charcoal-stripped fetal bovine serum (Sigma-Aldrich F6765), and 1% penicillin/streptomycin. HESC were used experimentally no more than 5 passages from purchase. HESC were cultured in 5% CO_2_ incubators at 37 °C. Routine mycoplasma testing was performed using a MycoAlert^™^ Mycoplasma Detection Kit (Lonza). Cell ancestry information (e.g., racial and ethnic background, age, gender identity) was not provided by the vendor although the cell ancestry may affect cellular behavior and response^48,49^.

#### Primary human endometrial epithelial cell culture.

We cultured primary human endometrial epithelial cells (EECs; LifeLine Cell Technology FC- 0078; Lot 03839; Caucasian Female Donor, 33 y.o., uterine prolapse) as per the manufacturer’s instructions in phenol red-free medium (LifeLine Cell Technology) and in 5% CO2 incubators at 37°C. EECs were used experimentally at two passages from receipt (cells cryopreserved by vendor at passage 3). Cells were routinely tested for mycoplasma contamination using the MycoAlert^™^ Mycoplasma Detection Kit (Lonza).

#### Swan71 culture.

For trophoblast experiments, we used Swan 71 cells derived from a 7-week first-trimester placenta; however, no additional donor information was provided^61^. Swan71 were cultured in a growth medium consisting of phenol red-free DMEM (SCS Cell Media Facility, UIUC) supplemented with 10% charcoal-stripped fetal bovine serum, 1% penicillin/streptomycin, and 500 ng/mL puromycin. Once passaged for experiments, the cells were cultured in phenol red-free DMEM, 2% charcoal-stripped fetal bovine serum, and 1% penicillin/streptomycin (Swan71 motility medium). Swan71 cells (passage 1 from receipt) were provided by Dr. Gabriela Dveksler (Uniformed Services University of Health Sciences, Bethesda, MD) and Dr. Gil Mor (Yale University School of Medicine, New Haven, CT) or purchased (Applied Biological Materials Inc. T0532).

#### 2D culture of human endometrial microvascular endothelial cells.

HEMEC cells were seeded into individual wells of a 6 well plate and cultured until confluent in endothelial cell medium. Cells were then fixed in formalin (Sigma-Aldrich), permeabilized for 15 minutes in 0.5% Tween20 (Fisher Scientific BP337), washed 3x5 minutes with 0.1% Tween20 solution (PBST), blocked with 2% Abdil (2% bovine serum albumin; Sigma Aldrich A4503 + 0.1% Tween20) for 1 hour, and stained with primary antibodies (1:200 CD31; Dako IS610 or 2.5 μg/mL von Willebrand Factor viii; Invitrogen MA5-14029) overnight at 4°C. 5x5 minute PBST washes were performed followed by staining with secondary antibody (1:500 Alexafluor 488 goat anti-mouse; Thermo Fisher A-11001) overnight at 4 °C. 5x5 minute PBST washes were performed, followed by staining with Hoechst (1:2000; Thermo Fisher H3570) for 10 minutes at room temperature. One final PBST wash was performed and cells were stored in PBST until imaged. Wells were imaged using a Leica DMI 4000 B Microscope (Leica Microsystems).

#### Matrigel tube formation assay.

100 μL of phenol red-free Matrigel (1.35 mg protein/well; Corning 356237) was pipetted into each well of a 96-well plate and polymerized in the incubator. 10,000 HEMECs were added per well and were cultured in an endothelial cell medium (*n* = 8 wells). Each well was imaged at 6 hours and 12 hours after seeding using a Leica DMI 4000 B Microscope (Leica Microsystems). The tubes fall apart after 24 hours of culture which is a known limitation of the Matrigel tube formation assay^62,63^.

#### Endometrial microvascular network hydrogel fabrication and maintenance.

HEMEC and HESC were passaged and encapsulated in GelMA hydrogels at 1:1, 1:2, and 2:1 endothelial:stromal cell ratios. The concentration of endothelial cells was kept consistent with each ratio at 500,000 HEMEC/mL, and the concentration of stromal cells was calculated from this value and the ratios. Hydrogels were cultured in 48 well plates for 7 days and maintained in endothelial cell medium with or without additional growth factors (±100 ng/mL recombinant human VEGF_165_; PeproTech 100-20) and hormones. The medium for hydrogel samples was replaced every 3 days (800 μL/well). The endogenous VEGF concentration in the endothelial cell medium was reported to be 2 ng/mL by the vendor (ScienCell). All experiments except those involving hormones used charcoal-stripped fetal bovine serum (Sigma-Aldrich F6765) instead of regular fetal bovine serum to decrease endogenous hormones in the base medium.

#### Microvascular network decidualization.

Decidualization of endometrial stromal cells in the microvascular networks was induced by culturing the microvascular network hydrogels in the presence of the following decidualization hormone cocktails added to the endothelial cell medium: (i) based on synthetic progesterone, 1 μM medroxyprogesterone acetate (MPA; Sigma-Aldrich M1629) + 0.5 mM 8-bromodenosine 3’,5’-cyclic monophosphate (8-Br-cAMP; Sigma-Aldrich B5386) or (ii) progesterone based, 0.5 mM dibutyryl cyclic AMP (dcAMP; Millipore Sigma 28745) + 10 nM estradiol (E2; Sigma-Aldrich E2758) + 100 nM progesterone (P4; Sigma-Aldrich P8783). Control hydrogels had no exogenous hormones added to the medium. Medium was replaced every 3 days, collected at days 3 and 6, and stored at − 80°C.

#### Microvascular network conditioned media effects on trophoblast motility.

Control and decidualized microvascular network hydrogels were cultured as described above. Media were collected during media changes on days 3 and 6 of culture, filtered, and stored at −20°C until use. Unconditioned, unspent endothelial cell medium was also collected for use as a control condition. Conditioned media from days 3 and 6 were pooled (all hydrogel samples and all days combined) prior to adding to spheroid cultures. Spheroid motility assays were performed as described previously by our group^59,60,64^. Briefly, Swan71 cells were cultured in flasks until 80-90% confluence and added to round bottom plates (Corning 4515; 4,000 cells/well) for 24-48 hours in the incubator until spheroids formed. Individual spheroids were encapsulated in GelMA hydrogels and maintained in 800 μL of medium (Swan71 motility medium, 50:50 Swan71 motility medium:endothelial cell medium, or 50:50 Swan71 motility medium:microvascular-conditioned medium) for 3 days. Each encapsulated spheroid was imaged daily on a Leica DMI 4000 B microscope (Leica Microsystems). The total outgrowth area was calculated using the measure tool in FIJI by averaging three traced measurements of the outgrowth area. Fold change was calculated by normalizing the outgrowth area to the initial spheroid area (day 0) using Eq. 1. A supplemental control experiment was performed to investigate how exogeneous decidualization hormones influenced trophoblast motility. Using the same methodology described above, we incubated encapsulated trophoblast spheroids without exogenous hormones (control) or with decidualization cocktails (1 μM MPA + 0.5 mM 8-Br-cAMP or 0.5 mM dcAMP + 10 nM E2 + 100 nM P4; Sigma-Aldrich P8783).


(1) $$Fold\;Change_{Day3}=\frac{Spheroid\;Area_{Day3}-Spheroid\;Area_{Day0}}{Spheroid\;Area_{Day0}}$$

#### Trophoblast-conditioned media effects on microvascular network hydrogels.

Swan71 were cultured in growth medium consisting of phenol red-free DMEM (SCS Cell Media Facility, UIUC) supplemented with 10% charcoal-stripped fetal bovine serum, 1% penicillin/streptomycin, and 500 ng/mL puromycin. Swan71 cells were cultured in a T75 culture flask and medium was collected at confluence, syringe filtered, and stored at −20°C until use. Fresh unconditioned growth medium was also collected for use as a control. Co-culture hydrogels were fabricated and cultured as described above in endothelial cell medium (control), a 50:50 ratio of endothelial cell medium to unconditioned Swan71 motility medium (media control), or 50:50 ratio endothelial cell medium to Swan71 conditioned medium. Hydrogels were stained, imaged, and analyzed as described above.

### Tri-culture of endometrial endothelial, stromal, and epithelial cells

Microvascular network hydrogel cultures were prepared as described above and cast into Ibidi μ-Slides Angiogenesis (10 μL prepolymer solution; Ibidi 81506). Polymerized gels were then coated with Collagen 1 (EMD Millipore 08-115MI) and Collagen 3 (EMD Millipore CC054) using microbial transglutaminase (mTg; Zedira T001)^55,65,66^. A 1:1 ratio of 0.5 mg/mL mTg and 10 μg/mL ECM protein (1:1 ratio Collagen 1 and Collagen 3) were combined, and 20 μL of this solution was pipetted onto hydrogels. Coated hydrogels were incubated for 1 hour in 5% CO2 incubators at 37 °C. A quick wash was performed using 20 μL of PBS. After the wash step, we seeded 200,000 EEC/cm^2^ onto hydrogels. We cultured tricultures for 7 days and subsequently stained them with CD31 and phalloidin (7 μL per 1000 μL solution) or cytokeratin 18 (CK18; 1:250, Cell Signaling 24E10) using the protocol described above. We then took Z-stack images of each gel from the top of the gel as far down as we could visualize. We took 1 Z-stack per gel (*n* = 2 gels per condition).

### Characterization of microvascular network cultures

#### Immunofluorescent staining.

On day 7 of culture, hydrogel samples (co-culture and tri-cultures) were fixed with formalin (Sigma-Aldrich) and washed three times with PBS. Hydrogels were permeabilized for 15 minutes in a 0.5% Tween20 (Fisher Scientific BP337) solution and washed 3x5 minutes in 0.1% Tween20 solution (PBST). Samples were blocked for 1 hour at room temperature in a 2% Abdil solution (2% bovine serum albumin; Sigma Aldrich A4503 + 0.1% Tween20) and subsequently incubated in primary antibody solution (1:200 CD31 Dako IS610 + 1:200 CD10 Invitrogen PA5-85875 or 1:200 anti-laminin Abcam ab11575 or 5 μg/mL ZO-1 Invitrogen #61-7300) overnight at 4 °C. 4x20 minutes washes with PBST were performed and then cultured in secondary antibody (1:500 Alexafluor 555 goat anti-rabbit Thermo Fisher A-21428 and/or 1:500 Alexafluor 488 goat anti-mouse Thermo Fisher A-11001) overnight at 4°C. Hydrogels were washed 4x20 minutes with PBST and then incubated for 30 minutes in Hoechst (1:2000; Thermo Fisher H3570). Samples were washed a final time in PBST and were stored in PBST until imaged.

#### Microscopy techniques.

Stained hydrogels were imaged using glass bottom confocal (In Vitro Scientific, D29-20-1-N) dishes on a DMi8 Yokogawa W1 spinning disc confocal microscope outfitted with a Hamamatsu EM-CCD digital camera (Leica Microsystems). Three 100 μm z-stacks with a 5 μm step size were taken for each hydrogel for 3 regions of interest (ROI) except for time course experiments and laminin/ZO-1 stained hydrogels (*n* = 2–3 hydrogels; *n* = 2 ROI per hydrogel). For day 14 and day 21 hydrogels, 1 ROI was imaged, which captured roughly 80–100% of the entire gel area. Fluorescent images were artificially brightened for figures but not for analysis.

#### Image analysis.

Images of the stained, microvascular network hydrogel samples were analyzed using a computational pipeline consisting of a FIJI macro and custom MATLAB algorithm^67,68^. This pipeline allows for 3D quantification of vessel networks across image z-stacks. Briefly, individual images of each z-stack were blurred, filtered, and binarized using a FIJI macro. Then, the binarized images were analyzed using a custom MATLAB algorithm that quantified total branches + endpoints, branch points, number of vessels, and total network length from skeletonized images. Examples of binarized z-slice images for each major experiment can be found in Supplemental Figure S8. Using Microsoft Excel, we then calculated the total network length / mm^3^, average branch length (network length / number of vessels), number of branches, and number of vessels for each sample.

To compute the degree of overlap between the CD31 signal and laminin/ZO-1 signal, CD31 and laminin/ZO-1 Z-stacks were binarized using the same FIJI macro listed above. Compressed copies of the Z-stacks were also created to match the size of the binarized images. Average pixel intensity was generated for both stains, and the degree of overlap was then calculated by multiplying the binarized matrix of microvascular networks by the binarized matrix of the proteins.

#### Cytokine array.

A Proteome Profiler Human Angiogenesis Array (R&D Systems ARY007) was used to determine relative levels of 55 angiogenesis-related proteins. Medium from the microvascular network cultures was collected at days 3 and 6 of culture, stored at −80°C until use, and pooled for analysis. 500 μL of medium was used for each day (1 mL total per cytokine array; *n* = 3 separate membranes per condition). The array was run as per the manufacturer’s instructions and imaged (4-minute exposure) using an Amersham ImageQuant 800 Fluor system (Cytiva). The pixel density of each array spot was quantified using FIJI. The negative control spot averages were subtracted from the pixel density of each sample, and then the pixel density of each sample was normalized to the pixel density of positive control spots.

#### STRING analysis.

Statistical analysis was performed to determine which of the 55 angiogenesis-related proteins were statistically significantly different between groups. The resultant analysis revealed 14 significant proteins. These 14 proteins were entered into the STRING (Search Tool for the Retrieval of Interacting Genes/Proteins) Database to determine known and predicted protein-protein interactions^69–71^. A network summary view was created using a medium confidence minimum required interaction score (0.400).

### Statistical analysis

OriginLab 2021b, RStudio, and GraphPad Prism10 were used for statistical analyses. Normality was determined via Shapiro-Wilkes and homoscedasticity was determined via Levene’s test or Bartlett’s Test. Data were analyzed using a one-way analysis of variance (ANOVA) and Tukey post hoc test (normal, homoscedastic), Welch’s ANOVA and Games-Howell post hoc test (normal, heteroscedastic), Kruskall-Wallis ANOVA and Dunn’s post hoc test (non-normal, homoscedastic), or Welch’s Heteroscedastic F Test with Trimmed Means and Winsorized Variances and Games-Howell post hoc test (non-normal, heteroscedastic). Significance was set as *p* < 0.05 and data are presented as mean ± standard deviation unless otherwise described. Each quantitative experiment used *n* = 3–6 hydrogels and followed these statistical protocols unless otherwise clarified. Plots were generated using OriginLab or Prism10.

## Supplementary Material

Supp Info

## Figures and Tables

**Table 1 |:** Relevance of cytokines to endometrial function

Protein	Relevance	References
Activin A	Expressed in decidualized stromal cells	45,46
Amphiregulin	Role in receptivity and blastocyst attachment	43
Angiogenin	Angiogenesis; Decidualization	72
Angiopoetin-1	Maintains vessel integrity and vascular remodeling	5,39
Endoglin	Endometrial receptivity	73
Endostatin/Collagen XVIII	Maturation and stabilization of vessels	41
Endothelin-1	Vasoconstriction; Myometrial contraction	6,74
FGF-1	Angiogenesis	39,40
IGFBP-2	Found in stroma; Abundant in secretory phase	75,76
PDGF	Angiogenesis; Stromal cell motility and proliferation	42
Pentraxin 3	Angiogenesis; Matrix Remodeling; Inflammation; Decidualization	77
Platelet Factor 4	Repair following menses	44
Prolactin	Decidualization	78
Serpin F1	Regulation of angiogenesis	79

## Data Availability

All data needed to evaluate the conclusions in the paper are present in the paper and the Supplementary Materials. Data can also be found in Dryad: https://doi.org/10.5061/dryad.p2ngf1vwn.
