# Supplementary material for: Endometrial decidualization status modulates endometrial microvascular complexity and trophoblast outgrowth in gelatin methacryloyl hydrogels
Source: NPJ Womens Health. Author manuscript; Available in PMC 2025 Jun 24. (PMC11259096; doi:10.1038/s44294-024-00020-4)
Supplement: Supp Info [file NIHMS2008432-supplement-Supp_Info.pdf]

Supplementary Materials for

Endometrial decidualization status modulates endometrial microvascular complexity and trophoblast outgrowth in gelatin methacryloyl hydrogels

Samantha G. Zambuto, Hannah Theriault, Ishita Jain, Cody Crosby, Ioana Pintescu, Noah Chiou, Michelle L. Oyen, Janet Zoldan, Gregory H. Underhill, Brendan A.C. Harley*, Kathryn B.H. Clancy*

*Co-corresponding author emails: bharley@illinois.edu, kclancy@illinois.edu

This PDF file includes:

Figs. S1 to S8

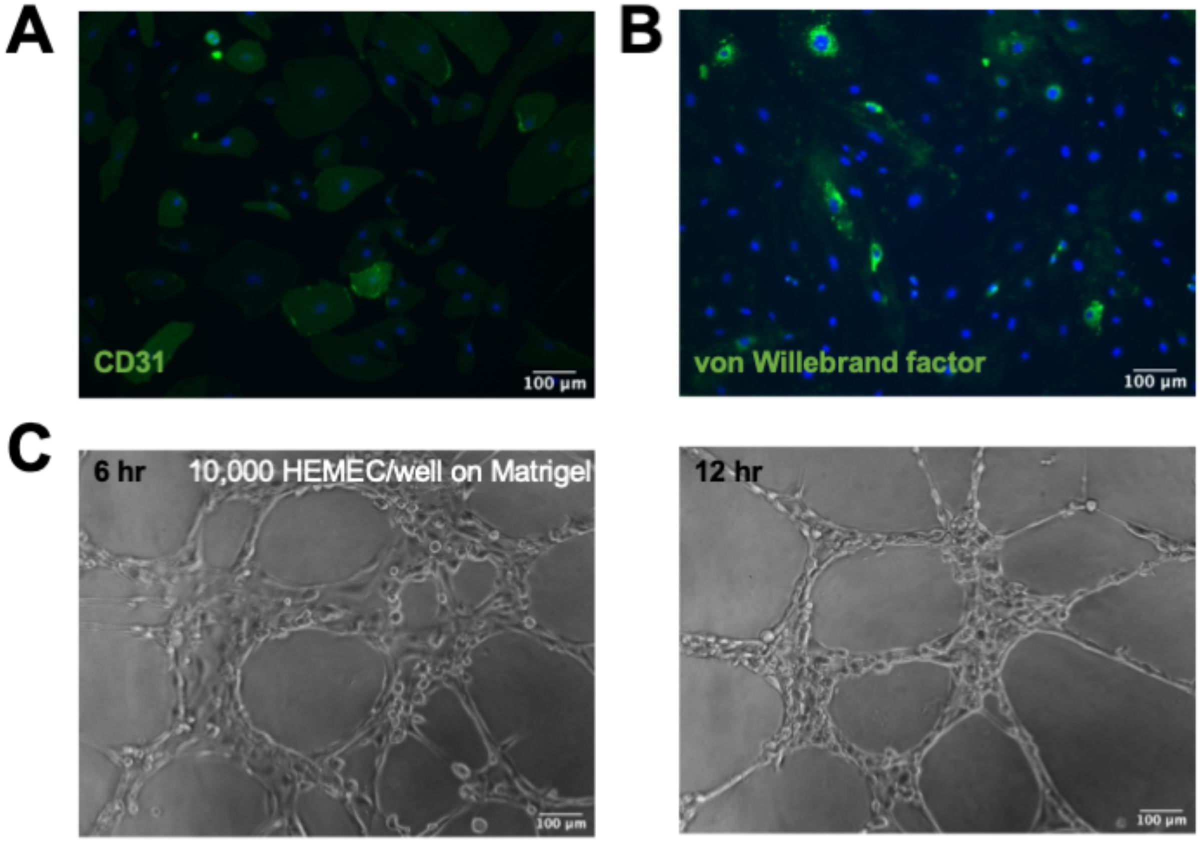

Fig. S1.

Angiogenic potential of human endometrial microvascular endothelial cells (HEMEC). HEMEC cultured on well plates express characteristic endothelial cell markers such as (A) CD31 (cluster of differentiation 31) and (B) von Willebrand factor. (C) HEMEC demonstrate the ability to form tubes on Matrigel. Scale bar: 100 μm.

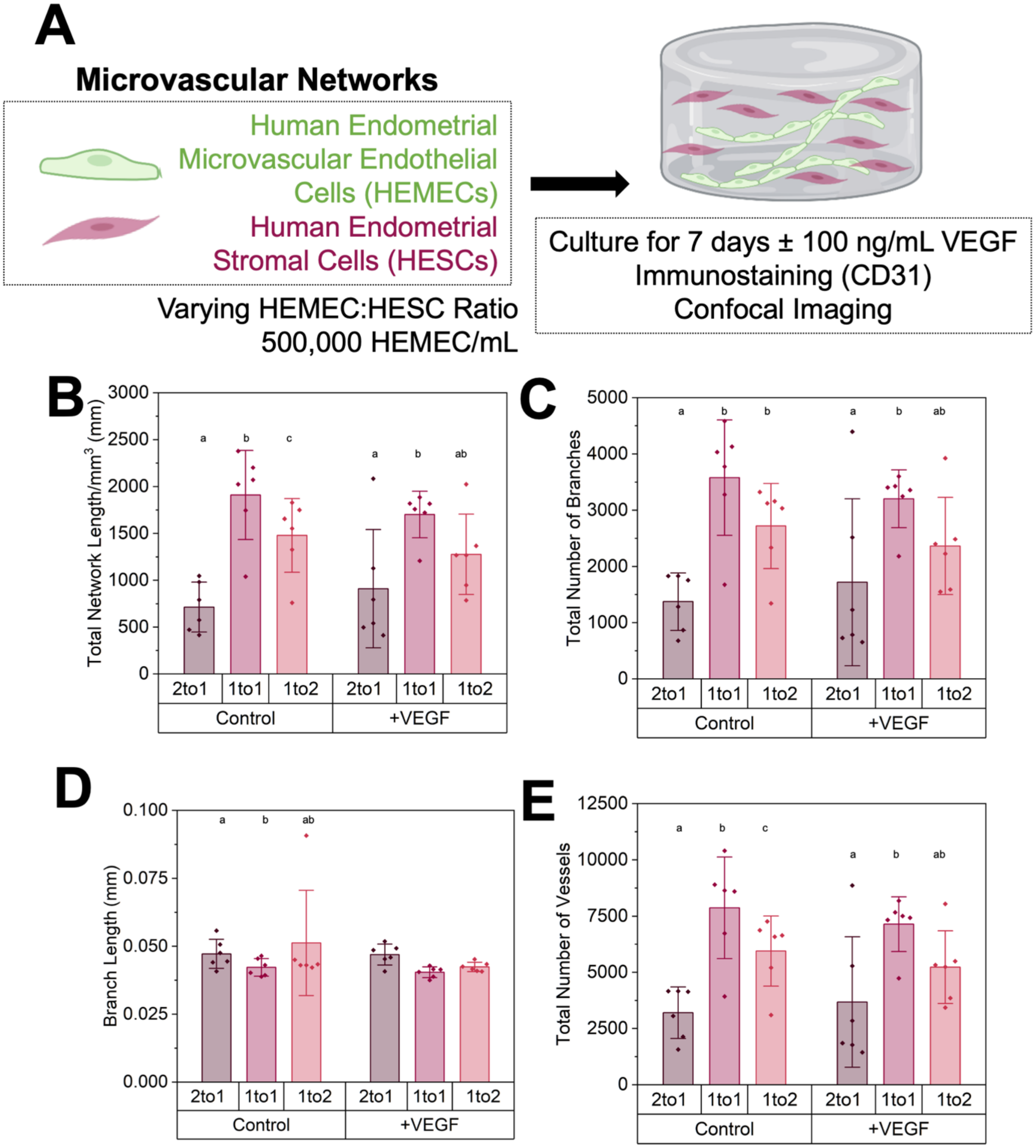

Fig. S2.

Optimization of an endometrial microvascular network. (A) Experimental summary. (B) Quantification of total vessel length per mm^3 , (C) total number of branches, (D) average branch length, and (E) total number of vessels for control and vascular endothelial growth factor (VEGF) samples (n=6 hydrogels per condition; 3 ROI imaged per gel and averaged) of varying endothelial to stromal cell ratios. Groups with different letters are statistically significantly different from each other. Data presented as mean ± standard deviation. Created with Biorender.com.

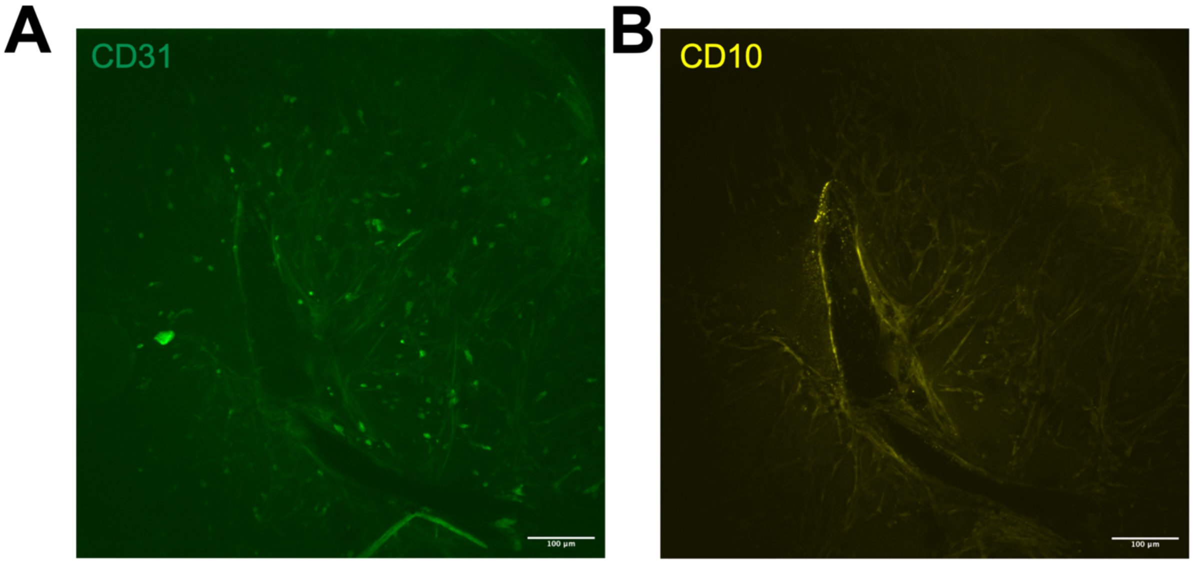

Fig. S3.
Characterization of endometrial microvascular networks. (A) Maximum intensity projections of Z-stacks of artificial endometrial perivascular niche hydrogel cultures (2:1 endothelial:stromal cell ratio stained for CD31 (HEMEC-endothelial cells) and (B) CD10 (HESC-stromal cells. Scale bars: 100 µm. Images artificially brightened for visualization using FIJI.

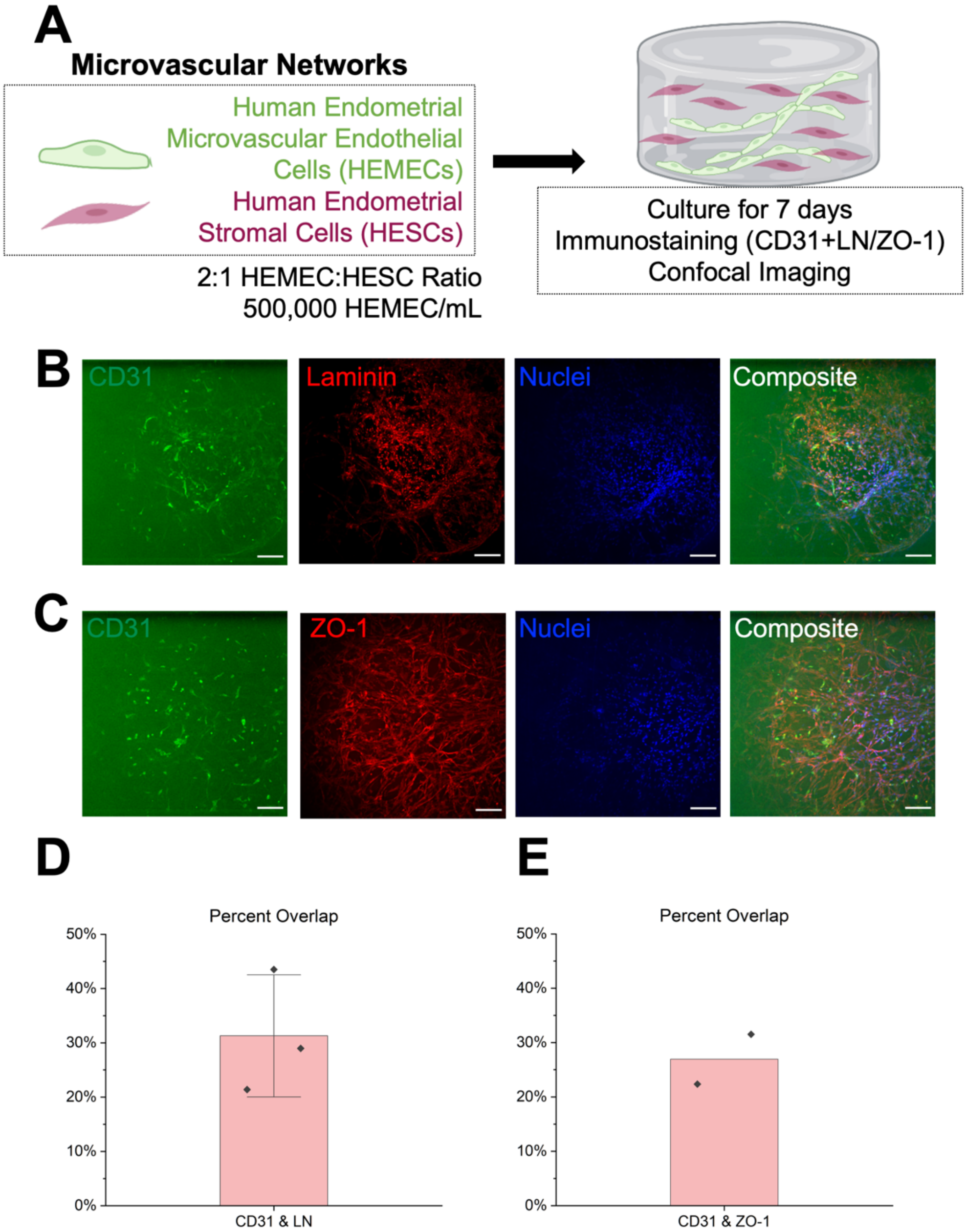

Fig. S4.

Characterization of extracellular matrix deposition and tight junction expression in endometrial microvascular networks. (A) Experimental summary. Maximum intensity projections of Z-stacks of artificial endometrial perivascular niche hydrogel cultures stained for CD31 (HEMEC-endothelial cells) and (B) laminin (C) and ZO-1. Green-CD31; Red-Laminin or ZO-1; Blue-Nuclei. Percent overlap was calculated between CD31 signal and (D) laminin and (E) ZO-1. n=2-3 hydrogels per condition. Data presented as mean ± standard deviation. 2 ROI imaged per gel. Scale bars: 100 μm. Images artificially brightened for visualization using FIJI. Created with Biorender.com.

A

Mean Pixel Density	Control	MPA+8-Br-cAMP	E2+P4+dcAMP
Activin A	246	4452	2859
ADAMTS-1	70	283	374
Angiogenin	1312	664	1489
Angiopoietin-1	4807	842	4011
Angiopoietin-2	22471	33062	35244
Angiostatin/Plasminogen	82	276	276
Amphiregulin	1025	10883	16458
Artemin	299	354	753
Coagulation Factor III	667	811	1437
CXCL16	879	542	690
DPPIV	2008	2989	1489
EGF	65343	67106	68178
EG-VEGF	416	489	603
Endoglin	377	434	875
Endostatin/Collagen XVIII	1334	1565	4021
Endothelin-1	1266	4229	8369
FGF acidic	741	3015	2646
FGF basic	11155	14635	13477
FGF-4	106	173	207
FGF-7	237	214	465
GDNF	143	311	937
GM-CSF	710	206	519
HB-EGF	506	475	1045
HGF	33197	8326	28412
IGFBP-1	595	1783	1266
IGFBP-2	50118	2654	12800
IGFBP-3	323	1284	2415
IL-1B	339	335	2770
IL-8	68128	70560	47970
LAP (TGFB1)	221	535	5659
Leptin	146	180	189
MCP-1	4899	119	313
MIP-1a	248	387	454
MMP-8	367	777	592
MMP-9	680	612	747
NRG1-B1	335	895	720
Pentraxin 3 (PTX3)	21859	3627	7978
PD-ECGF	479	572	845
PDGF-AA	748	1095	1797
PDGF-AB/PDGF-BB	181	1167	2397
Persephin	975	886	1152
Platelet Factor 4 (PF4)	135	640	921
PIGF	325	918	920
Prolactin	124	296	568
Serpin B5	261	455	414
Serpin E1	42192	53811	48065
Serpin F1	31364	1790	11788
TIMP-1	52847	62420	61022
TIMP-4	596	414	697
Thrombospondin-1	59355	48578	61095
Thrombospondin-2	482	1027	1138
uPA	49561	64154	68833
Vasohibin	479	458	315
VEGF	40786	55978	45471
VEGF-C	142	207	280

Maximum	70560
Median	879
Minimum	70

B

Normalized Mean Pixel Density	Control	MPA+8-Br-cAMP	E2+P4+dcAMP
Activin A	0.0041	0.0725	0.0459
ADAMTS-1	0.0012	0.0046	0.0059
Angiogenin	0.0221	0.0108	0.0237
Angiopoietin-1	0.0812	0.0138	0.0640
Angiopoietin-2	0.3808	0.5448	0.5662
Angiostatin/Plasminogen	0.0014	0.0045	0.0043
Amphiregulin	0.0172	0.1788	0.2642
Artemin	0.0050	0.0057	0.0120
Coagulation Factor III	0.0111	0.0133	0.0229
CXCL16	0.0147	0.0088	0.0108
DPPIV	0.0339	0.0488	0.0237
EGF	1.1055	1.0983	1.0906
EG-VEGF	0.0070	0.0080	0.0096
Endoglin	0.0063	0.0072	0.0140
Endostatin/Collagen XVIII	0.0224	0.0255	0.0645
Endothelin-1	0.0214	0.0706	0.1353
FGF acidic	0.0127	0.0491	0.0423
FGF basic	0.1878	0.2390	0.2145
FGF-4	0.0018	0.0028	0.0033
FGF-7	0.0040	0.0035	0.0074
GDNF	0.0024	0.0051	0.0146
GM-CSF	0.0119	0.0034	0.0081
HB-EGF	0.0085	0.0078	0.0163
HGF	0.5625	0.1360	0.4443
IGFBP-1	0.0100	0.0293	0.0204
IGFBP-2	0.8496	0.0435	0.2092
IGFBP-3	0.0054	0.0209	0.0380
IL-1B	0.0057	0.0054	0.0430
IL-8	1.1524	1.1563	0.7807
LAP (TGFB1)	0.0037	0.0088	0.0877
Leptin	0.0024	0.0030	0.0030
MCP-1	0.0822	0.0019	0.0049
MIP-1a	0.0041	0.0063	0.0072
MMP-8	0.0061	0.0131	0.0094
MMP-9	0.0114	0.0101	0.0118
NRG1-B1	0.0057	0.0150	0.0116
Pentraxin 3 (PTX3)	0.3704	0.0597	0.1291
PD-ECGF	0.0081	0.0095	0.0135
PDGF-AA	0.0126	0.0180	0.0287
PDGF-AB/PDGF-BB	0.0030	0.0196	0.0386
Persephin	0.0164	0.0142	0.0184
Platelet Factor 4 (PF4)	0.0023	0.0107	0.0148
PIGF	0.0054	0.0150	0.0145
Prolactin	0.0021	0.0048	0.0090
Serpin B5	0.0044	0.0075	0.0065
Serpin E1	0.7154	0.8826	0.7707
Serpin F1	0.5310	0.0293	0.1892
TIMP-1	0.8937	1.0247	0.9757
TIMP-4	0.0101	0.0069	0.0111
Thrombospondin-1	1.0040	0.7992	0.9791
Thrombospondin-2	0.0081	0.0168	0.0180
uPA	0.8398	1.0517	1.1016
Vasohibin	0.0080	0.0076	0.0050
VEGF	0.6904	0.9192	0.7278
VEGF-C	0.0024	0.0034	0.0045

Maximum	1.1563
Median	0.0123
Minimum	0.0012

Fig. S5. Cytokine array data. **A.** Raw mean pixel density values. **B.** Mean pixel density values normalized to positive control spots.

Proteins	Normal	Homoscedastic	Statistical Test	Significance	P-Value	PostHoc
Activin A	Yes	Yes	One Way ANOVA	Yes	0.00032216	MPA-B, P4-C, Control-A
ADAMTS-1	Yes	Yes	One Way ANOVA	No	0.13928088	-
Angiogenin	Yes	Yes	One Way ANOVA	Yes	0.02877175	P4-B, Control-AB, MPA-A
Angiopoietin-1	Yes	Yes	One Way ANOVA	Yes	0.00011577	Control-A, P4-A, MPA-B
Angiopoietin-2	Yes	Yes	One Way ANOVA	No	0.15683049	-
Angiostatin/Plasminogen	Yes	Yes	One Way ANOVA	No	0.15094149	-
Amphiregulin	Yes	Yes	One Way ANOVA	Yes	0.00092357	P4-B, MPA-B, Control-A
Artemin	Yes	Yes	One Way ANOVA	No	0.08793302	-
Coagulation Factor III	Yes	Yes	One Way ANOVA	No	0.39112243	-
CXCL16	Yes	Yes	One Way ANOVA	No	0.59122583	-
DPPIV	Yes	Yes	One Way ANOVA	No	0.17167048	-
EGF	No	Yes	Kruskal-Wallis	No	0.875170	-
EG-VEGF	Yes	Yes	One Way ANOVA	No	0.52736328	-
Endoglin	Yes	Yes	One Way ANOVA	Yes	0.04611137	All-A
Endostatin/Collagen XVIII	Yes	Yes	One Way ANOVA	Yes	0.01733964	P4-B, MPA-A, Control-A
Endothelin-1	No	Yes	Kruskal-Wallis	Yes	0.03899022	Control-A, P4-B, MPA-AB
FGF Acidic	Yes	Yes	One Way ANOVA	Yes	0.00170789	MPA-B, P4-B Control-A
FGF Basic	No	Yes	Kruskal-Wallis	No	0.73263247	-
FGF-4	Yes	Yes	One Way ANOVA	No	0.32356892	-
FGF-7	Yes	Yes	One Way ANOVA	No	0.21461955	-
GDNF	Yes	Yes	One Way ANOVA	No	0.42457538	-
GM-CSF	Yes	Yes	One Way ANOVA	No	0.3643465	-
HB-EGF	Yes	Yes	One Way ANOVA	No	0.60389266	-
HGF	Yes	Yes	One Way ANOVA	No	0.27355501	-
IGFBP-1	No	Yes	Kruskal-Wallis	No	0.05090583	-
IGFBP-2	Yes	Yes	One Way ANOVA	Yes	0.00041646	Control-A, P4-B, MPA-B
IGFBP-3	No	Yes	Kruskal-Wallis	No	0.05090583	-
IL-1B	No	Yes	Kruskal-Wallis	No	0.73263247	-
IL-8	Yes	Yes	One Way ANOVA	No	0.417633	-
LAP (TGFB1)	No	Yes	Kruskal-Wallis	No	0.09915102	-
Leptin	Yes	Yes	One Way ANOVA	No	0.87586041	-
MCP-1	Yes	Yes	One Way ANOVA	No	0.09033782	-
MIP-1a	Yes	Yes	One Way ANOVA	No	0.5876582	-
MMP-8	Yes	Yes	One Way ANOVA	No	0.54288516	-
MMP-9	Yes	Yes	One Way ANOVA	No	0.92173073	-
NRG1-B1	No	Yes	Kruskal-Wallis	No	0.06646292	-
Pentraxin 3 (PTX3)	Yes	Yes	One Way ANOVA	Yes	0.00017908	Control-A, P4-B, MPA-B
PD-ECGF	Yes	Yes	One Way ANOVA	No	0.1810274	-
PDGF-AA	Yes	Yes	One Way ANOVA	Yes	0.04183885	P4-B, MPA-AB, Control-A
PDGF-AB/PDGF-BB	No	Yes	Kruskal-Wallis	No	0.05090583	-
Persephin	Yes	Yes	One Way ANOVA	No	0.85485408	-
Platelet Factor 4 (PF4)	Yes	Yes	One Way ANOVA	Yes	0.02629441	P4-B, MPA-AB, Control-A
PIGF	Yes	Yes	One Way ANOVA	No	0.06548898	-
Prolactin	Yes	Yes	One Way ANOVA	Yes	0.03153584	P4-B, MPA-AB, Control-A
Serpin B5	Yes	Yes	One Way ANOVA	No	0.43676163	-
Serpin E1	No	Yes	Kruskal-Wallis	No	0.17669445	-
Serpin F1	Yes	Yes	One Way ANOVA	Yes	6.28E-06	Control-A, P4-B, MPA-C
TIMP-1	Yes	Yes	One Way ANOVA	No	0.10180256	-
TIMP-4	Yes	Yes	One Way ANOVA	No	0.31298174	-
Thrombospondin-1	Yes	Yes	One Way ANOVA	No	0.11408469	-
Thrombospondin-2	Yes	Yes	One Way ANOVA	No	0.14951137	-
uPA	Yes	Yes	One Way ANOVA	No	0.05267839	-
Vasohibin	Yes	Yes	One Way ANOVA	No	0.64923927	-
VEGF	No	Yes	Kruskal-Wallis	No	0.06081006	-
VEGF-C	Yes	Yes	One Way ANOVA	No	0.30144158	-

Fig. S6. Statistical analysis of 55 cytokines. Green: Yes. Red: No. One Way ANOVA post hoc: Tukey post hoc test. Kruskal-Wallis post hoc: Dunn’s post hoc test.

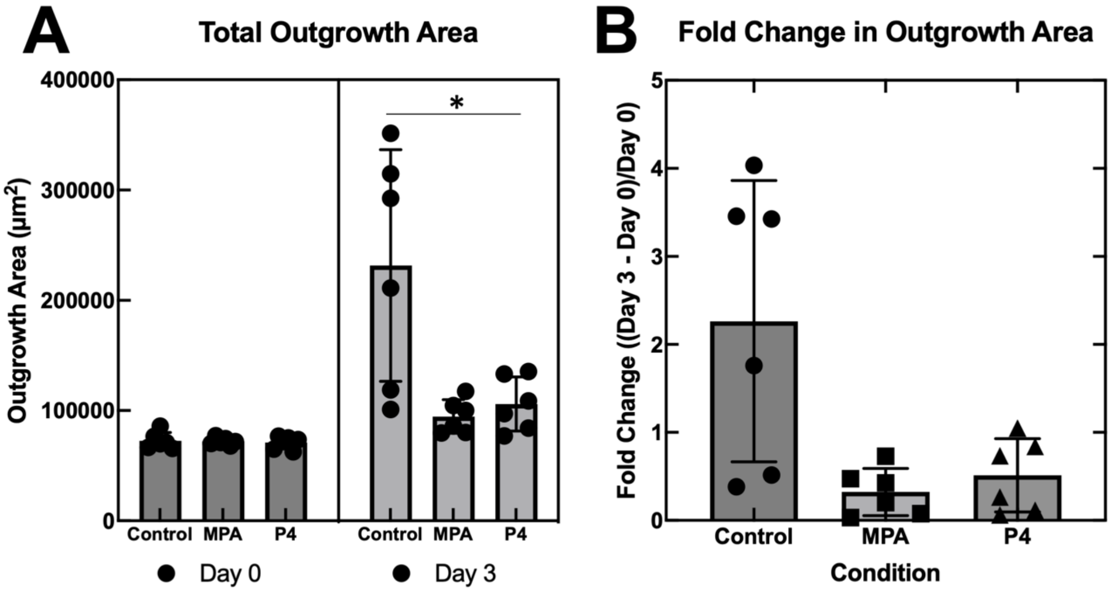

Fig. S7. Swan71 trophoblast motility in the presence of hormones. **A.** Quantification of total outgrowth area (µm^2) and **B.** fold change in outgrowth area at Day 3 compared to Day 0 (encapsulation) for control condition (no factors added) and media conditions supplemented with decidualization cocktails containing MPA or P4. Data presented as mean ± standard deviation (n=6 samples per condition). MPA: medroxyprogesterone acetate, P4: progesterone. *: $p < 0.05$ for Welch's ANOVA but Dunnett's T3 Multiple Comparison's Test posthoc analysis revealed no significant differences between groups.

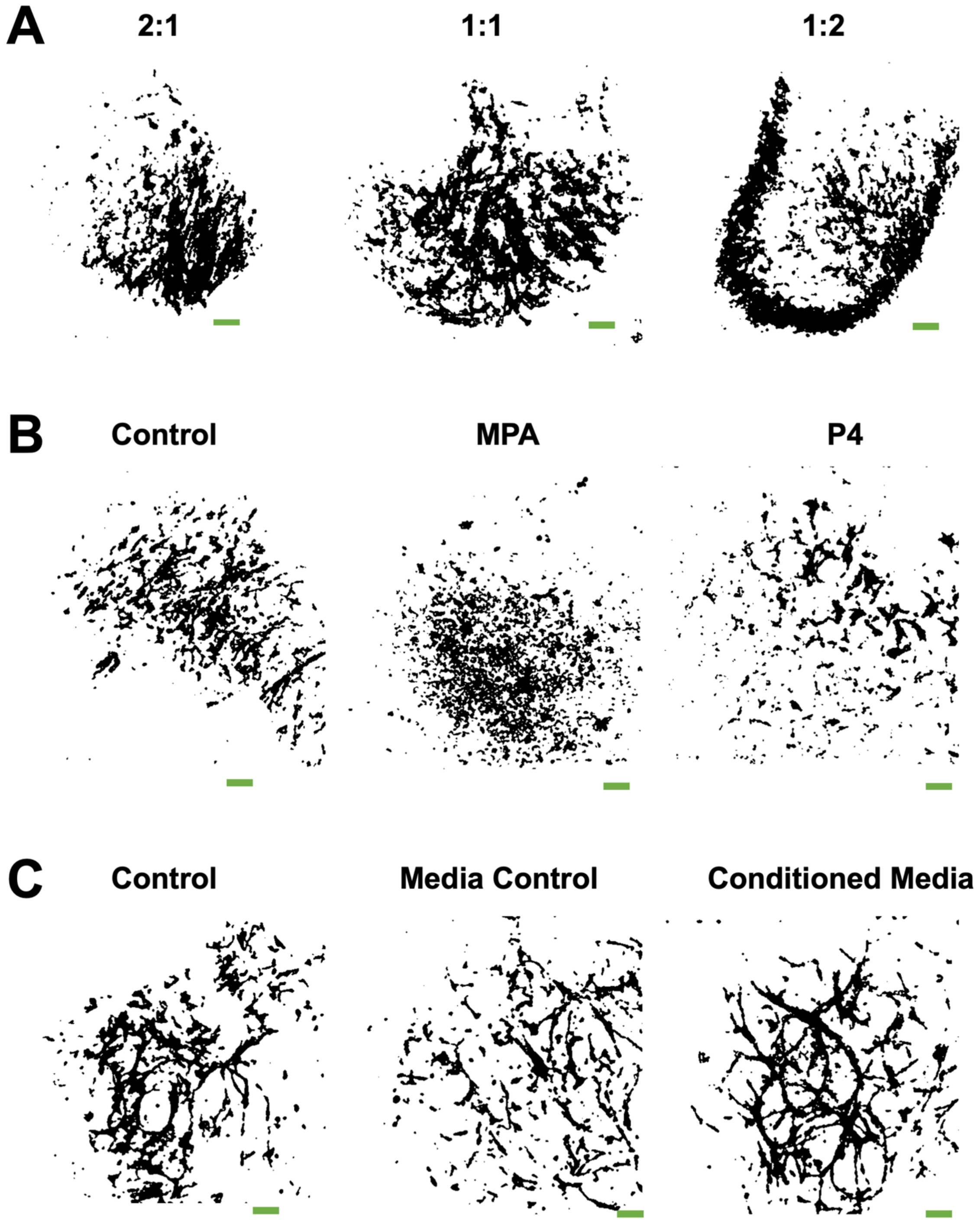

Fig. S8. Representative binarized image slices of microvascular networks. Each image is an example of a binarized Z-slice from Z-stacks of CD31 stained HEMEC cells in hydrogels. **A.** Day 7 binarized images for varying ratios HEMEC:HESC. **B.** Day 7 binarized images for decidualized and control samples. **C.** Day 7 binarized images of control, media control, conditioned medium samples. Scale bar: 100 μm.
